# Dissection of mammalian orthoreovirus µ2 reveals a self-associative domain required for binding to microtubules but not to factory matrix protein µNS

**DOI:** 10.1371/journal.pone.0184356

**Published:** 2017-09-07

**Authors:** Catherine Eichwald, Jonghwa Kim, Max L. Nibert

**Affiliations:** 1 Department of Microbiology & Immunobiology, Harvard Medical School, Boston, Massachusetts, United States of America; 2 Institute of Virology, University of Zurich, Zurich, Switzerland; 3 Laboratory of Gastroenterology, Samsung Medical Center, Seoul, Republic of Korea; SRI International, UNITED STATES

## Abstract

Mammalian orthoreovirus protein μ2 is a component of the viral core particle. Its activities include RNA binding and hydrolysis of the γ-phosphate from NTPs and RNA 5´-termini, suggesting roles as a cofactor for the viral RNA-dependent RNA polymerase, λ3, first enzyme in 5´-capping of viral plus-strand RNAs, and/or prohibitory of RNA-5´-triphosphate-activated antiviral signaling. Within infected cells, μ2 also contributes to viral factories, cytoplasmic structures in which genome replication and particle assembly occur. By associating with both microtubules (MTs) and viral factory matrix protein μNS, μ2 can anchor the factories to MTs, the full effects of which remain unknown. In this study, a protease-hypersensitive region allowed μ2 to be dissected into two large fragments corresponding to residues 1–282 and 283–736. Fusions with enhanced green fluorescent protein revealed that these amino- and carboxyl-terminal regions of μ2 associate in cells with either MTs or μNS, respectively. More exhaustive deletion analysis defined μ2 residues 1–325 as the minimal contiguous region that associates with MTs in the absence of the self-associating tag. A region involved in μ2 self-association was mapped to residues 283–325, and self-association involving this region was essential for MT-association as well. Likewise, we mapped that μNS-binding site in μ2 relates to residues 290–453 which is independent of μ2 self-association. These findings suggest that μ2 monomers or oligomers can bind to MTs and μNS, but that self-association involving μ2 residues 283–325 is specifically relevant for MT-association during viral factories formation.

## Introduction

The M1 genome segment of mammalian orthoreovirus (MRV) encodes the 83-kDa μ2 protein, one of five protein components of the MRV core particle [1–4]. The core, with ~20 copies of μ2 [5], is released into the cytoplasm as the “payload” of cell entry [6, 7]. Once in the cytoplasm, it mediates transcription, 5´-capping and export of full-length plus-strand RNAs templates by the ten double-stranded RNA (dsRNA) genome segments [4, 8]. These transcripts are then used for translation of the MRV proteins or as templates for full-length minus-strand RNA synthesis and packaging to generate new dsRNA segments within newly assembling core particles. At least some of the newly assembled cores also produce plus-strand transcripts, either before or instead of undergoing outer-capsid assembly to become infectious virions, substantially by amplifying the levels of MRV transcripts and proteins produced in infected cells [9–12].

Although the precise roles of μ2 in RNA synthesis remain uncertain, the purified protein acts as a nucleoside triphosphatase (NTPase) as well as an RNA 5´-triphosphatase (RTPase) [13]. In the same lines, there is evidence showing that the μ2-encoding M1 genome segment is a genetic determinant of MRV strain differences in *in vitro* NTPase activities of cores, resulting in critical dependence of viral replication [14, 15]. The M1 genome segment is also the genetic determinant of strain differences in *in vitro* transcription yields and responses to different temperatures by cores [16] as well as in sensitivity of MRV growth to mycophenolic acid, which decreases cellular pools of GTP [17]. A temperature-sensitive mutant that renders μ2 defective at supporting MRV genome synthesis in cells suggests that newly synthesized μ2 may also be a component of the replicase complex or otherwise involved at an early step in viral replication [18]. Results from knockdown of μ2 expression by RNA interference are consistent with this conclusion [19, 20]. Together, these findings suggest roles for μ2 in RNA synthesis in both mature (transcribing) and nascent (assembling and genome-replicating) cores. Interaction of μ2 with the viral RNA-dependent RNA polymerase (RdRp) λ3 has been demonstrated with the two purified proteins [13]. Purified μ2 also binds RNA [21], which may be further necessary for its roles in cores.

Perhaps relating to its roles in nascent cores, μ2 is a component of viral factories [22, 23], which are the sites of MRV genome replication and particle assembly in cells. The MRV nonstructural protein μNS forms the matrix of these factories, and μ2 has been shown to associate with μNS in infected or co-transfected cells [23, 24]. A minimal region of μNS both necessary and sufficient for the μ2 association has been defined [14, 24, 25], but the regions of μ2 involved in μNS association remain unknown.

MRV factories can be observed by light microscopy and have two basic morphologies, globular or filamentous [23, 26–29]. When expressed in the absence of other MRV proteins, μNS forms factory-like structures (FLS) that are morphologically similar to globular factories. Remarkably, the μ2 protein of certain MRV strains, including Type 1 Lang (T1L) in co-expression with μNS redirect the FLS to microtubules (MTs), generating filamentous structures [24]. In fact, the single expression of μ2 protein associate and stabilize MTs in cells [23], and purified T1L μ2 showed to co-sediment with purified MTs *in vitro* [13]. The globular morphology of factories formed by a few other MRV strains [23, 28, 29] has been attributed to point mutations in μ2, as in the case of our lab’s version of strain Type 3 Dearing (T3D^N^), which contains a the substitution of a Ser by Pro at residue 208 (S208P). When expressed in the absence of μNS, T3D^N^ μ2 shows a reduced capacity to associate with MTs and tends to forms small aggregates [23, 29, 30]. Complementation of μ2 *in trans* after silencing of core-transcribed M1 mRNAs by RNA interference has provided direct evidence that MT association by μ2 is essential for MRV growth in at least some settings [19]. However, the regions of μ2 involved in MT association remain unknown. Interestingly, the residue 208 mediates repression of IFN-ß signaling, modulating MRV induction of IFN-ß in mice cardiac myocytes [31]. IFN-ß regulation by μ2 has been associated with the presence of a conserved sequence present among MRV strains, denoted as immunoreceptor tyrosine-based activation motif (ITAM). This motif is present in the μ2 amino acid (aa) region 118–134, and it is related to the activation of NF-κB and the recruitment of Syk kinase intermediate into the viral factories [32]. Also, T1L μ2 can retain IRF9 in the nucleus, a protein associated with the innate immune response suppression of T1L strains but not of T3D strains [33, 34]. The presence of a nuclear localization signal motif in μ2 residues 99 to 110 [14] could also play a role in this pathway, as it may be a requirement for μ2 nuclear import and further suppression of the host innate immune response.

More complex phenotypes associated with MRV infections are also genetically influenced by M1/μ2. These phenotypes include (i) the capacity of MRV T1L to grow more efficiently than T3D in primary myocardial cells [35]; (ii) the ability of T1L, but not T3D, to grow effectively in endothelial and Madin-Darby canine kidney cells by favoring progeny assembly, with viral fitness analysis suggesting that cell tropism variations can be due to a critical role related to aa 347 [36–38]; (iii) the greater capacity of MRV variant 8B to cause myocarditis in mice, correlating phenotypes in cultured cardiac myocytes [39–41]; (iv) the largest capacity of T1L than T3D to grow and cause lesions in the liver, coupled with the stronger virulence of T1L with respect to T3D, in severe-combined-immunodeficient mice [42]. These M1-influenced differences in growth efficiency and virulence between MRV strains suggest that μ2 may interface with host factors to influence the outcome of infection in particular cells or tissues. This aspect could be accomplished in several ways if: (i) the RTPase activity of μ2 prohibits RNA-5´-triphosphate-activated antiviral signaling pathways [43–45] or (ii) the MT-association activity of μ2 affects MRV growth, spread, or effects on cells [19, 23].

Given that there are many unsolved questions regarding the roles of μ2 in MRV growth and effect of host cells, we were interested in functionally dissecting the determinants present in the μ2 protein. In this study, we used fluorescence microscopy as a tool for gauging the associations of μ2 with MTs and itself, as well as the relationship between these two activities. Following the same methodology, we establish the minimal requirement of μ2 to associate with the matrix forming protein, μNS into FLS. The results obtained expand our understanding of the functional organization of μ2 by identifying N-terminal regions of the protein that are essential for all three activities.

## Materials and methods

### Cells, viruses, and antibodies

CV-1 (African green monkey kidney fibroblast, M.L. Nibert own collection, HMS) cells were cultured in Dulbecco’s modified Eagle’s medium (Invitrogen) supplemented with 10% fetal bovine serum (HyClone) and 10μg/ml gentamicin (Invitrogen). MRV strains T1L and T3D^N^ were derived from the Bernard N. Fields lab. Rabbit polyclonal antisera specific for μ2 and μNS were used as described previously [23, 46]. Mouse antibody (MAb) anti-acetylated a-tubulin (clone 6-11B-1) and mouse MAb anti-α-tubulin (clone B-5-1-2) were obtained from Sigma-Aldrich. Rabbit polyclonal anti-tubulin (H-300) and mouse MAb anti-GFP (B-2) were obtained from Santa Cruz Biotechnology, Inc. Rabbit polyclonal anti-GFP (ab290) was obtained from Abcam. Mouse MAb HA.11 was obtained from Covance; goat anti-rabbit immunoglobulin G (IgG) conjugated to Alexa 594, goat anti-mouse IgG conjugated to Alexa 594, and goat anti-mouse IgG conjugated to Alexa 488, were obtained from Molecular Probes, Invitrogen.

### Proteolytic digestion and N-terminal sequencing of partially purified μ2

Baculovirus-based expression and partial purification of μ2(T1L) were performed as described previously [13]. Proteolytic treatment was carried out at room temperature in a 10-μl volume with 5 μg of μ2 and 0.5 μg of Thermolysin (Sigma-Aldrich) in digestion buffer (20 mM Tris acetate (pH 8.5), 50 mM KCl, 5 mM MgCl_2_, 10% glycerol, 2 mM β-mercaptoethanol). The reaction was stopped by addition of 0.5 μl of 0.5 M EDTA pH 8.0. For N-terminal sequencing, samples were loaded onto 10% deionized polyacrylamide mini gels. The gel was pre-run for 10 min at 80 mA with 2 mM thioglycolic acid (Fisher Scientific) in the upper buffer (125 mM Tris (pH 6.8), 0.1% SDS). Samples were denaturated by mixing with gel sample buffer (final concentrations: 125 mM Tris (pH 8.0), 10% sucrose, 1% SDS, 2% 2-mercaptoethanol, 0.01% bromophenol blue) and incubated for 30 min at 65°C. After this treatment, the samples were loaded and separated at 100 V for stacking gel and at 300–350 V through the resolving gel. Proteins were transferred to Immobilon-PSQ polyvinylidene fluoride membrane (Millipore) at 4°C for 50 min at 80 V. The membrane was washed in distilled water for 10 min, stained with Coomassie brilliant blue R-250 (Sigma-Aldrich) for 5 min, and destained twice in 50% methanol for 5 min and twice in water for 5 min. The membrane was air dried and mailed to Midwest Analytical, Inc., for N-terminal sequencing via Edman degradation.

### Oligonucleotides and plasmid constructions

The oligonucleotides were obtained from Invitrogen and are listed in S1 and S2 Tables. A detailed description of the plasmid constructions is provided in the supporting information (S1 File).

### Fluorescence microscopy

CV-1 cells (5 × 10^5^/well) growing in six-well plates (9.6 cm^2^/well) containing round glass coverslips (diameter, 18 mm) were transfected with 4 μg of plasmid and 10 μl of Lipofectamine 2000 (Invitrogen), according to manufacturer's instructions. At 20 h post transfection (hpt), the cells were fixed for 10 min in phosphate-buffered saline (PBS) (137 mM NaCl, 3 mM KCl, 8 mM Na_2_HPO_4_, 1 mM KH_2_PO_4_ (pH 7.5)) containing 2% paraformaldehyde (PFA) or when indicated, for 3 min in cold methanol at -20°C, permeabilized for 5 min in PBS containing 0.1% Triton X-100, and blocked in PBS containing 1% bovine serum albumin for 30 min. All steps were performed at room temperature. For immunofluorescence, primary and secondary antibodies were diluted in PBS containing 1% bovine serum albumin and incubated for 45 min at room temperature in a humid chamber. Nuclei were stained with 70 nM 4,6-diamino-2-phenylindole (DAPI) (Molecular Probes). Cells were mounted in Prolong Gold (Molecular Probes) and images were acquired using a Nikon Eclipse TE 2000-U fluorescence microscope or confocal laser-scanning microscope (Leica, DM 5500 Q) equipped with a 63 X 1.3 oil objective. Data were analyzed with Leica Application Suite (Mannheim, Germany) and the Imaris software package (Bitplane, Switzerland). Collected images were processed and optimized with Photoshop (Adobe Systems) or with ImageJ Version 1.42q (National Institutes of Health, USA). For counts, at least 100 cells were individually analyzed for each phenotype. Images were prepared for publication using PowerPoint (Microsoft) software.

### Cytoplasmic platform assay for protein-protein associations

The assay was performed as described previously [47–50]. Briefly, CV-1 cells growing at 5 × 10^5^ cells/well in six-well plates were transfected as described above with 10 μl of Lipofectamine 2000, 4 μg of “fish” plasmid and 1 μg of “bait” plasmid. At 20 hpt, cells were fixed in PBS containing 2% PFA for 10 min at room temperature. Nuclei were stained with 70 nM DAPI. Cells were mounted in Prolong Gold and observed in a Nikon Eclipse TE 2000-U fluorescence microscope. Collected images were processed and optimized with Photoshop or with ImageJ version 1.42q. Cells were scored as positive or negative concerning the localization of “fish” protein to the FLS platforms formed by “bait” protein.

### Pull down assay

For each experimental point, 3x10^6^ CV-1 cells were infected with recombinant vaccinia virus strain VVT7.3 [51] [MOI, 3 pfu/cell] for 1 h at 37°C and then transfected with 9 μg of DNA plasmid using Lipofectamine 2000 transfection reagent (Thermo Fisher Scientific) according to the manufacturer instructions. At 15 hpt, media was replaced by complete media containing 10 μM MG132 (Sigma-Aldrich) and incubated for 2 h at 37°C. Before lysis, cells were washed once with PBS, incubated with 600μM DSP (dithiobis(succinimidyl propionate), Thermo Fisher Scientific) in PBS for 15 min on ice and washed three times with 50 mM Tris/HCl pH 8.0, 150 mM NaCl. Cells were lysed in TNN buffer (100 mM Tris pH 8.0, 250 mM NaCl, 0.5% NP-40 and cOmplete protease inhibitor cocktail (Roche, Switzerland)) for 10 min on ice and centrifuged at 15000 x g for 7 min at 4°C. The supernatant (cellular extract) was recovered and mixed with 50 μl of PerfectPro Ni-NTA agarose (5 PRIME, Germany) equilibrated in 25 mM imidazole in PBS for 2 h at 4°C in a wheel. The flow through was recovered by centrifuging at 3000 x g for 2 min at 4°C. The resin was washed with 10 volumes of 35mM imidazole in PBS and eluted by incubation for 30 min in a wheel at 4°C with 100μl of 250 mM imidazole in PBS. Samples (cellular extract, flow through and elution) were analyzed by immunoblotting as previously described [52].

### Sequence alignments

Alignments of μ2-homolog proteins were performed using the program T-Coffee [53] at http://www.ebi.ac.uk/t-coffee/. The following sequences were analyzed (GenBank accession number listed for each): MRV T1L μ2 (AF461682), MRV Type 2 Jones μ2, (AAK54567), MRV T3D μ2 (AF461683), MRV Type 1 Clone 29 μ2 (AAR96289), MRV T1 Netherlands 1984 μ2 (AAR96290), MRV Type 2 Simian Virus 59 μ2 (AAR96292), MRV Type 3 Clone 44 μ2 (AAR96294), ARV 138 μA (AY557188), ARV 176 μA (AY557189), ARV S1133 μA (AY639610), ARV 918 μA (AY639617), ARV Muscovy duck reovirus S14 μA (ABJ80882), AqRV grass carp reovirus (AF450324), AqRV Golden shiner reovirus (AF403402), and AqRV golden ide reovirus (AF450324). All available sequences with unique variations in the region aligning with μ2 residues 290–307 are shown in the discussion.

## Results

### Protease-hypersensitive region near residue 280 in MRV μ2 protein

**Fig 1:**
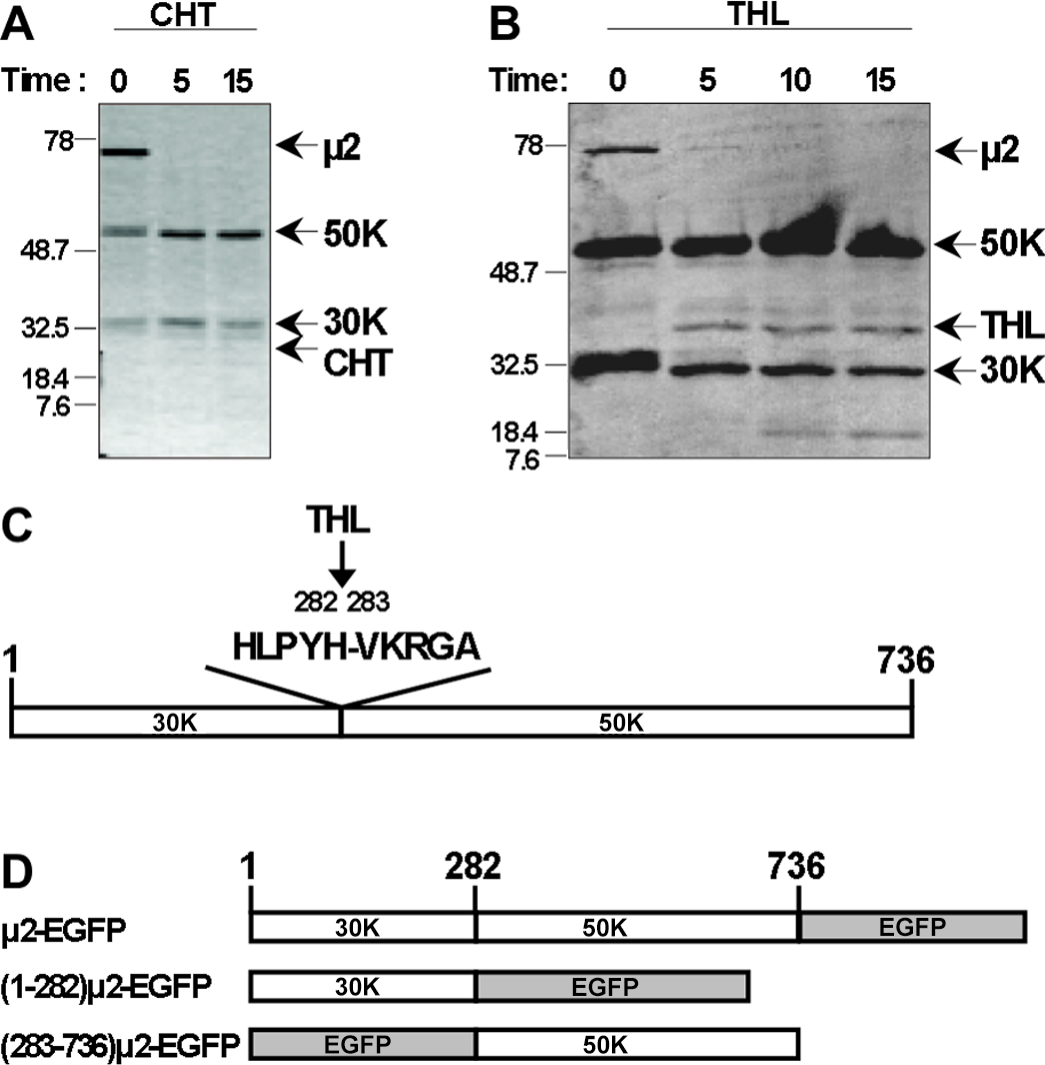
Protease-hypersensitive site in μ2. Partially purified T1L μ2 was treated with (A) 40 μg/ml chymotrypsin (CHT) or (B) 50 μg/ml thermolysin (THL) at room temperature for the indicated times (in minutes). The proteins were separated in in SDS-PAGE, followed by staining in gel with Coomassie blue. Arrows indicate the positions of full-length μ2, major proteolytic fragments (approximate molecular weights, 50 kDa (50K) and 30 kDa (30K), and the respective protease. The positions of molecular weight markers (kDa) are indicated at left. (C) Schematic representation of μ2 concluded from the N-terminal sequencing of the major THL-generated fragments. The 30K fragment yielded no N-terminal sequence, consistent with a blocked N-terminus. The 50K fragment yielded sequence Val-Lys-Arg-Gly-Ala (VKRGA), corresponding to μ2 residues 283–287 and consistent with THL cleavage. (D) Schematic representation of T1L μ2 full-length, 30K (residues 1–282) and 50K (residues 283–736) fused to an EGFP tag. Not to scale.

After partial purification and storage at 4°C [13], the 83-kDa μ2 protein of MRV T1L was slowly degraded into two fragments, ~50,000-M_r_ (50K) and ~30,000-M_r_ (30K), as assessed by SDS-PAGE (Fig 1A and 1B, 0-time point). Degradation was tentatively ascribed to small amounts of contaminating proteases in the μ2 preparation. Subsequent treatment with chymotrypsin or thermolysin enriched for fragments of similar sizes to those arising during storage (Fig 1A and 1B). When the two fragments from thermolysin digestion were subjected to N-terminal sequencing, the 30K fragment was found to be blocked, but the 50K fragment yielded sequences His-Val-Lys-Arg-Gly and Val-Lys-Arg-Gly-Ala, corresponding to aa 282–287 of T1L μ2. The first sequence is consistent with cleavage by a chymotrypsin-like protease after Tyr281, while the second sequence is compatible with cleavage by Thermolysin before Val283 (Fig 1C). These results suggested that the 30K and 50K fragments were likely the complementary N- and C-terminal portions of μ2 (Fig 1C), possibly reflecting two discrete domains of this protein. Based on these findings, we generated a series of new plasmid constructs for the study of protein association and distribution within the cell. These expression plasmids were engineered to express a full-length T1L μ2 (aa 1–736), its putative 30K portion (aa 1–282), or its putative 50K portion (aa 283–736), in each case fused with EGFP [54] (Fig 1D).

### MT-association by an N-terminal portion of EGFP-tagged μ2

**Fig 2:**
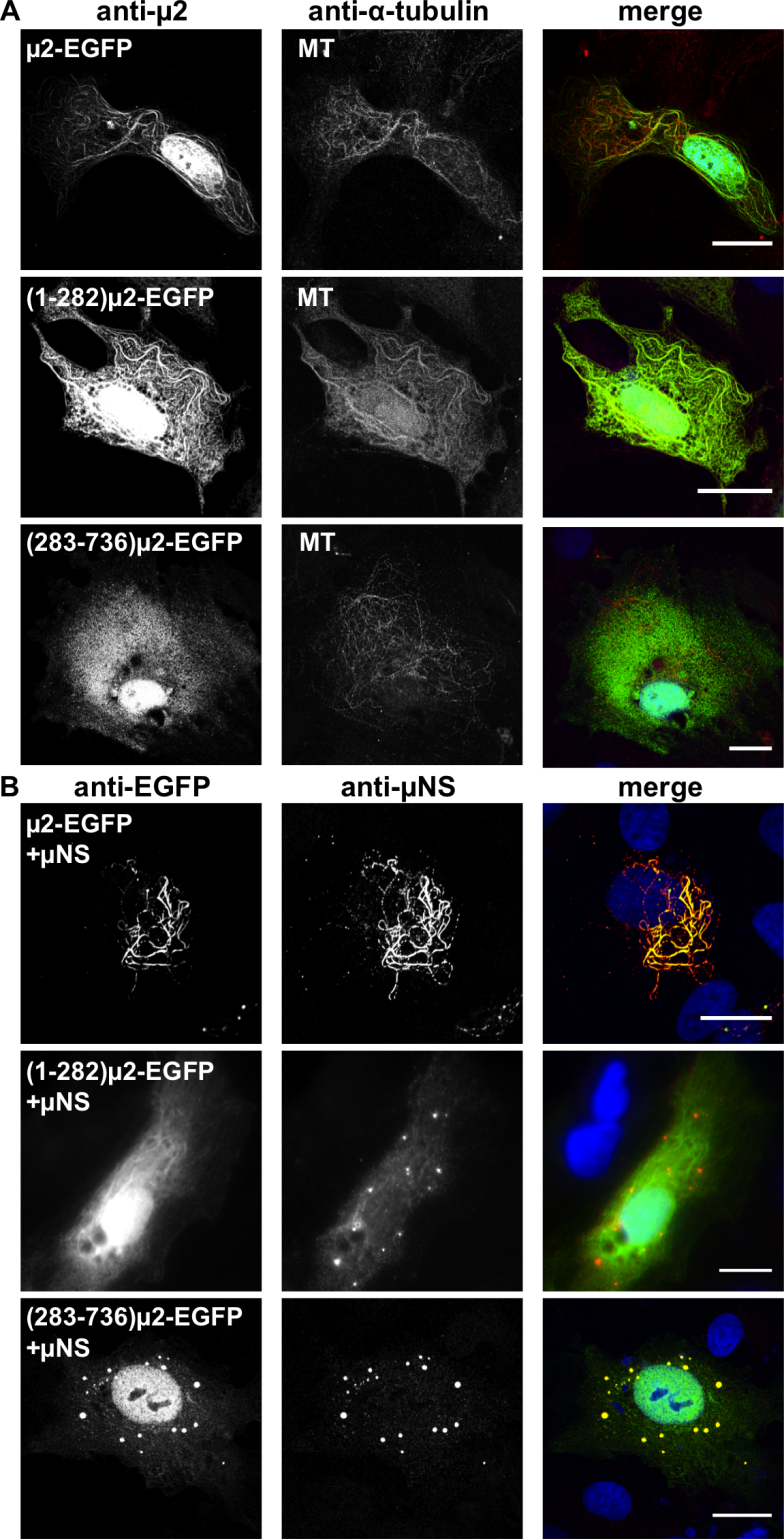
Cellular distribution of T1L μ2 full-length, aa regions (1–282) and (283–736) fused to EGFP. (A) Confocal immunofluorescence of CV-1 cells expressing T1L μ2 full-length or both aa regions (1–282) or (283–736) fused to EGFP. At 20 hpt, the cells were methanol fixed and immunostained for the detection of μ2 (specific polyclonal anti-μ2 serum, green) (left column) and MTs (mouse mAb anti-alpha tubulin, red) (middle column). The merged images are shown in the right column. Nuclei are stained with DAPI (blue). Scale bar is 20 μm. (B) Confocal immunofluorescence of CV-1 cells co-expressing T1L μNS with T1L μ2 full-length or both aa region (1–282) or (283–736) fused to EGFP. At 20 hpt, the cells were methanol fixed and immunostained for the detection of μ2 (mouse mAb anti-EGFP, green) (left column) and μNS (specific polyclonal anti-μNS serum, red) (middle column). The merged images are shown in the right column. Nuclei are stained with DAPI (blue). Scale bar is 20μm.

We first tested the μ2-EGFP fusion proteins for MT-association. After transient expression in CV-1 cells, both μ2-EGFP and (1–282)μ2-EGFP exhibited filamentous distributions (Fig 2A), consistent with MT-association as previously shown for non-tagged T1L μ2 [23]. Additionally, this μ2 filamentous distribution co-localized with MTs (Fig 2A) or acetylated-MTs (S1A Fig), and was not observed after treatment with the MT-depolymerizing drug nocodazole (S1B Fig). In marked contrast, EGFP-(283–736)μ2 showed no MT-association and was instead diffusely distributed within the cytoplasm (Fig 2A and S1A Fig). These findings suggest that some portion of μ2 aa 1–282 is both necessary and sufficient for MT-association in cells (i.e., μ2 aa 283–736 are dispensable for this activity).

### μNS association by a C-terminal portion of EGFP-tagged μ2

We next tested the μ2-EGFP fusion proteins for association with MRV factory-matrix protein μNS in FLS induced by μNS [24]. After transient co-expression in CV-1 cells, μ2-EGFP and EGFP-(283–736)μ2 were both positive for the μNS association, whereas (1–282)μ2-EGFP was negative (Fig 2B). In the case of μ2-EGFP, the distribution of μNS was filamentous; reflecting that μ2-EGFP recruited μNS to MTs as previously shown for non-tagged μ2 [24]. In the case of EGFP-(283–736)μ2, and in contrast with the lack of MT-association, we observed that μNS was found in globular FLS to which EGFP-(283–736)μ2 was recruited. As previously shown for non-tagged μ2 from MRV T3D clones that exhibit limited MT-association at 37°C [24, 30]. In the case of (1–282)μ2-EGFP, a third distinct pattern was observed since (1–282)μ2-EGFP exhibited filamentous distribution, reflecting MT-association while μNS localized separately, to globular FLS, consistent with the lack of μNS association by (1–282)μ2-EGFP. All the inspected cells presented an identical pattern when co-expressing of μ2-EGFP or its deletion mutants with μNS. These findings suggest that some portion of μ2 aa 283–736 is both necessary and sufficient for μNS association in FLS thereby further suggesting that MT-association and μNS association can be mapped to distinct, respective regions of μ2.

Additionally, pull-down assay was implemented to assess the interaction of μ2-EGFP, (1–282)μ2-EGFP or EGFP-(283–736)μ2 with μNS by adding a histidine tag at the EGFP of the μ2-EGFP derived proteins (S2 Fig). The results show that the three proteins were able to bind μNS despite the fact that (1–282)μ2-EGFP-histidine tagged did not localize in FLS, suggesting that a cytosolic soluble portion of these two proteins interact but without localizing in FLS.

### Smallest contiguous region of EGFP-tagged μ2 that mediates MT-association

**Fig 3:**
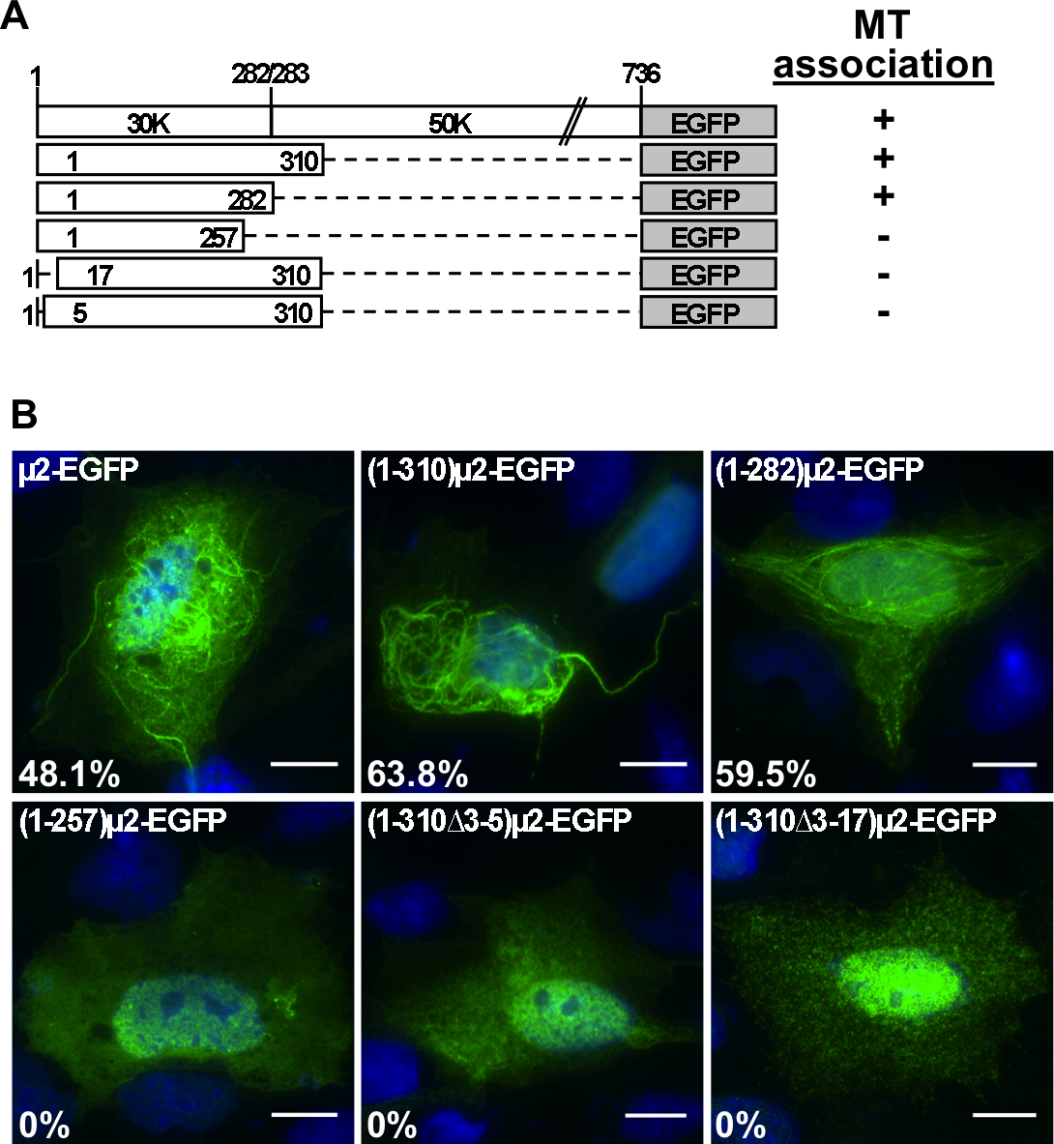
T1L μ2 region 1–282 fused to EGFP is necessary for MT-association. (A) Schematic representation of μ2 N-terminal deletion mutants fused to EGFP constructs (not to scale). Positive (+) or negative (-) for the formation of filamentous phenotype is indicated at right. (B) At 20 hpt, CV-1 cells expressing μ2 full-length or its deletion mutant fused to EGFP, as specified in each panel, were methanol fixed and immunostained for the detection of μ2 (mouse mAb anti-EGFP, green) and nuclei stained with DAPI (blue). The percentage of positive EGFP-tagged protein forming filamentous structures is indicated in each lower left corner. Scale bar is 10 μm.

Additional constructs were evaluated to define the minimal MT-association region of EGFP-tagged μ2. One set of constructs were designed to express even shorter N-terminal fragments, with progressively longer deletions from the C-terminus of the putative 30K region. Upon transient expression in CV-1 cells, however, even the next-shorter fragment, (1–257)μ2-EGFP, was negative for MT-association (Fig 3A and 3B). This result suggests that some portion of μ2 aa 258–282 is critical for MT-association. In this set of experiments, we also tested (1–310)μ2-EGFP, which was also positive for MT-association and in a similar fraction of transfected cells as μ2-EGFP and (1–282)μ2-EGFP (Fig 3A and 3B). Another set of constructs was designed to express EGFP fusions that lacked N-proximal residues of μ2. Because μ2 is modified by removal of Met1 and acetylation of Ala2 [55], we retained those residues in these new deletion mutants. By doing so, we ensure the acetylation of Ala in the deletion mutants as assumed by NetAcet prediction method [56]. Interestingly, however, fusions lacking μ2 aa 3–5 or 3–17, (1–310Δ3–5)μ2-EGFP and (1–310Δ3–17)μ2-EGFP, were negative for MT-association, as observed after transient expression in CV-1 cells (Fig 3A and 3B). These results suggest that an N-proximal region of μ2 is also important for MT-association. In summary, μ2 aa 1–282 constitute the smallest contiguous region of EGFP-tagged μ2 we have shown to be sufficient for MT-association, and sequences near each end of the 1–282 region appear to be essential for this activity.

### MT association by HA-tagged μ2

**Fig 4:**
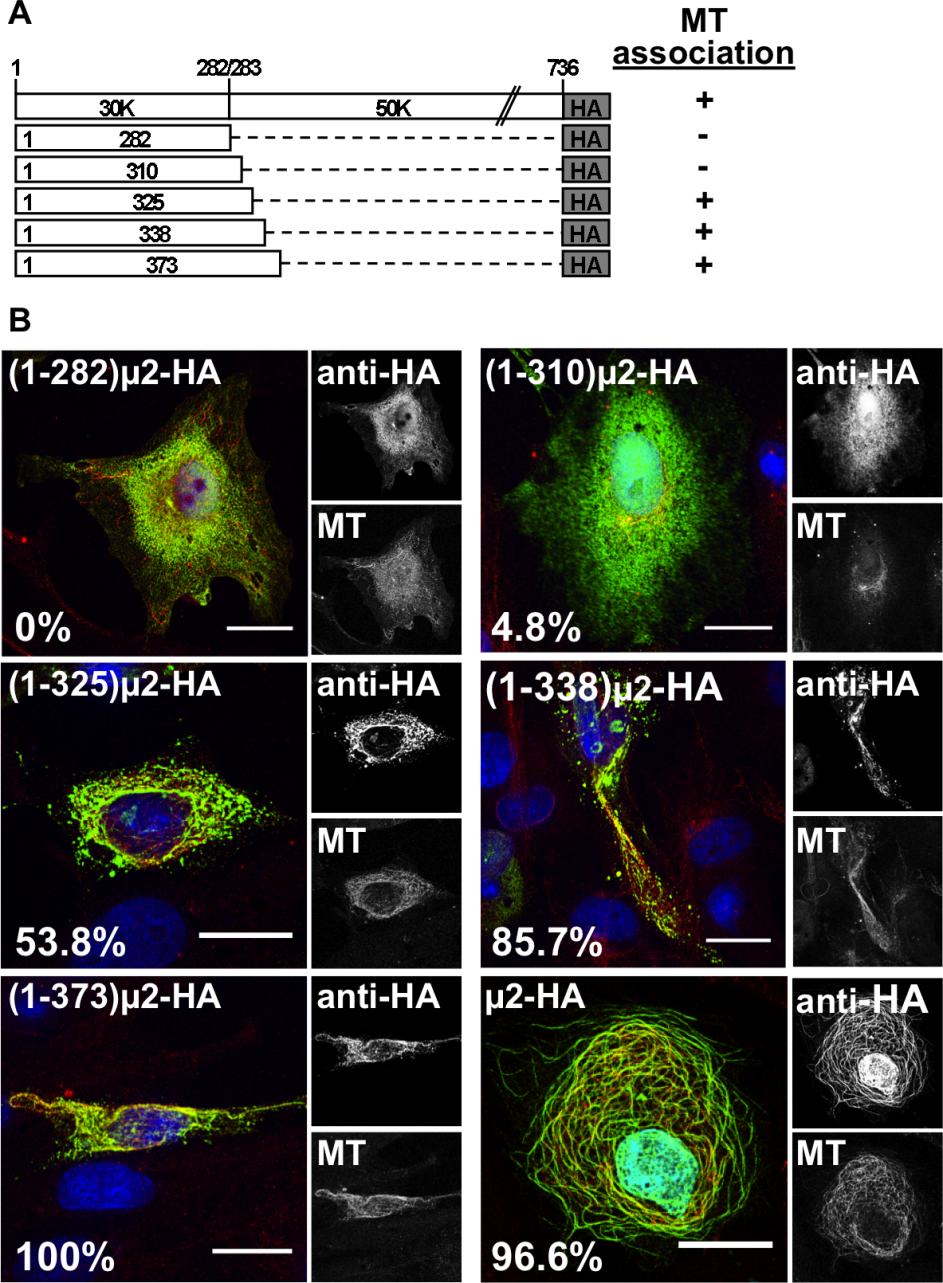
μ2 region 1 to 373 fused to HA tag is necessary for MT-association. (A) Schematic representation of T1L μ2 N-terminal deletion mutants fused to an HA tag (not to scale). Positive (+) or negative (-) MT-association phenotype is indicated at right. (B) Confocal immunofluorescence of CV-1 cells expressing μ2 or its deletion mutants with HA tag. At 20 hpt, cells were methanol fixed and immunostained for the detection of μ2 or its deletion mutants (mouse mAb anti-HA, green) (right upper panel) and MTs (specific polyclonal anti-alpha tubulin serum, red) (right lower panel). A Merged image is shown at the left of each panel. Nuclei are stained with DAPI (blue). Scale bar is 20 μm. The percentage of positive cells HA-tagged proteins associating to MTs is indicated at the left bottom of each merged image.

A caveat, however, is that EGFP weakly dimerizes [57] and might, therefore, display a self-association activity, possibly consequential for MT-association in cells, which is typically provided by a region of μ2 beyond aa 282 (see below for results consistent with this caveat).To evaluate the μ2 region that may be sufficient for MT-association in the absence of a self-associating tag like EGFP in the previous experiments, we tested several versions of the μ2 N-terminal region tagged with influenza virus HA epitope (Fig 4A). Following transient expression in CV-1 cells, (1–282)μ2-HA and (1–310)μ2-HA were, surprisingly, negative for MT-association (Fig 4B). In contrast, (1–325)μ2-HA, (1–338)μ2-HA, and (1–373)μ2-HA were all positive, with (1–373)μ2-HA showing MT association in a similar fraction of transfected cells as μ2-HA in the same experiment (Fig 4B). Based on these results, we revised our previous conclusion that some portion of μ2 aa 1–325 is necessary and sufficient for MT-association in cells in the absence of a self-associating protein like EGFP. Moreover, the apparent capacity of EGFP, which oligomerizes in cytosolic localization [58], to substitute for the 283–325 region of μ2 in allowing MT-association by (1–282)μ2-EGFP suggested that residues in the 283–325 region may be involved in μ2 self-association. This self-association may, in turn, be important for MT-association. Residues in the 325–373 region of μ2, which appeared to enhance MT-association by the HA-tagged proteins, might also participate in μ2 self-association. Also notable is that none of the HA-tagged μ2 proteins, except full-length μ2-HA, were positive for μNS association (see below), suggesting that a region of μ2 beyond aa 373 is required for the μNS association.

### Point mutations in μ2 that abrogate MT-association

**Fig 5:**
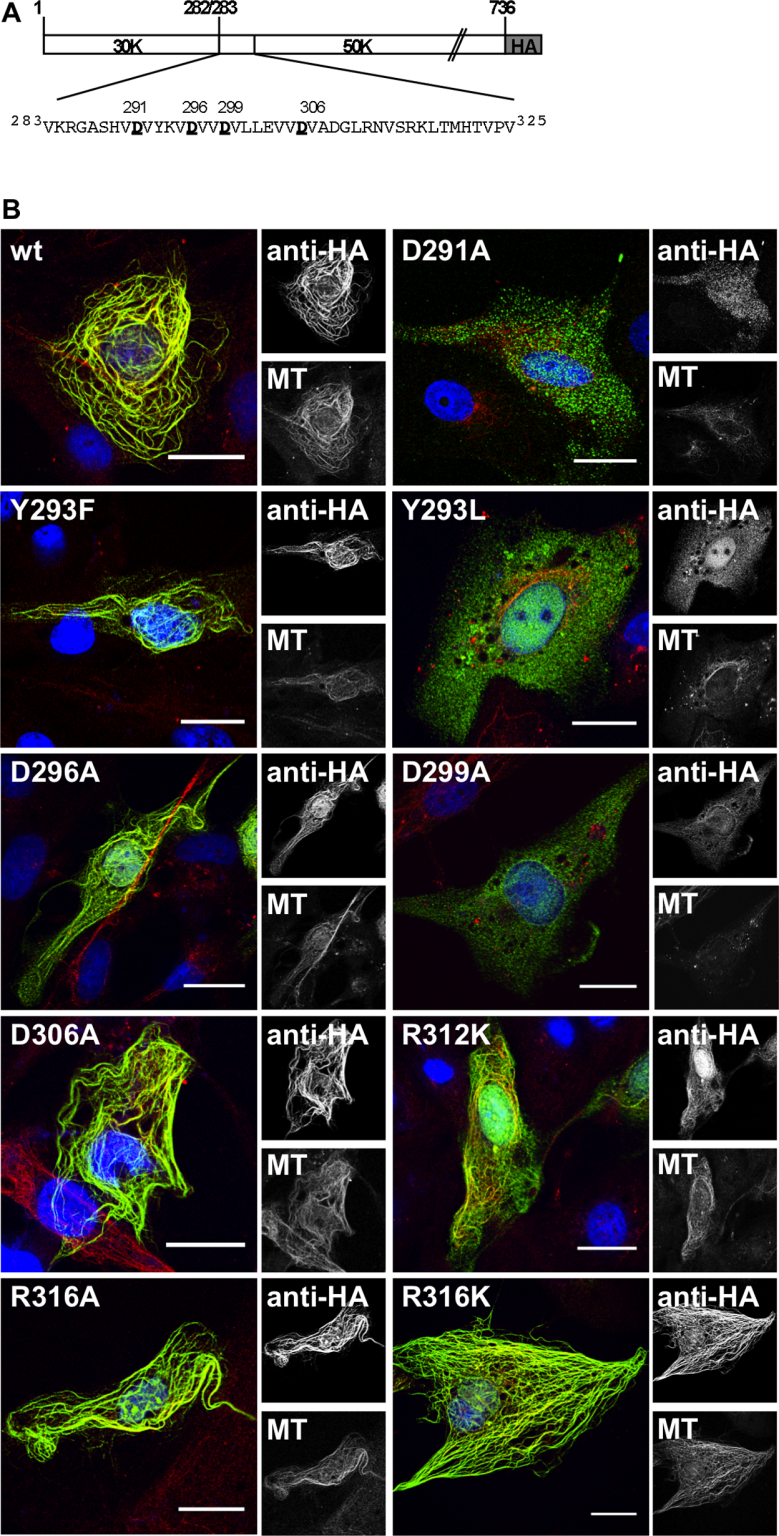
Point mutations in a repetitive motif in μ2 region, 283 to 325, abrogate MT-association. (A) Schematic representation of the amino acidic sequence in T1L μ2 region 283 to 325. The Asp in each Val-Asp-Val motif is underlined. In the scheme, the μ2 is fused at the C-terminus to an HA tag. (B) Confocal immunofluorescence of CV-1 cells expressing μ2-HA wild type or containing the specified point mutation. At 20 hpt, cells were methanol fixed and immunostained for detection of μ2 (mouse mAb anti-HA, green) (right upper panel) and MTs (specific polyclonal anti-alpha tubulin serum, red)(right lower panel). A Merged image is shown at the left of each panel. Nuclei are stained with DAPI (blue). Scale bar is 20 μm.

The putative self-association region of μ2, residues 283–325, contains four Val-Asp-Val repeats in residues 290–307 (Fig 5A). We hypothesized that these repeats might be involved in a mechanism encompassing 283–325 region that contributes to MT-association. To test this hypothesis, we engineered single Ala substitutions for residues Asp291, Asp296, Asp299, and Asp306 in the full-length μ2-HA. When transiently expressed in CV-1 cells, both μ2(D296A) and μ2(D306A) show a cytosolic filamentous distribution that is consistent with MT-association. Remarkably, when expressing μ2(D291A) or μ2(D299A), a cytosolic homogenous distribution was observed with a negative co-localization to the MT-network (Fig 5B). Notably, all four of these mutants remained positive for association with MRV factory-matrix protein μNS (S3 Fig and Table 1). Furthermore, other μ2 mutations, Y293F, R312K, S315A, R316A, and R316K, were also examined in the setting of full-length μ2, and all expressed proteins showed filamentous distribution and remained positive for association with both MTs (Fig 5B and Table 1) and μNS (S3 Fig). In the case of mutant μ2(Y293L), it was negative for MT-association (Fig 5B) but remained positive for association with the μNS association in globular FLS (S3 Fig). Thus, only three single-site substitutions within the 283–325 region of μ2, D291A, Y293L, and D299A, were found to abrogate MT-association, and none of these affected μNS association (Table 1).

**Table 1:** Summary of associations by μ2-HA and specified point mutations.

Plasmid-expressed μ2-HA protein^a^	MT-association^b^	μNS association^c^	μ2 self-association^d^
WT	+	+, F	+
D291A	-	+, G	-
Y293F	+	+, F	NT
Y293L	-	+, G	-
D296A	+	+, F	NT
D299A	-	+, G	-
D306A	+	+, F	NT
R312K	+	+, F	NT
S315A	+	+, F	NT
R316K	+	+, F	NT
R316A	+	+, F	NT

^**a**^ μ2-HA containing wild-type (WT) μ2 or the specified μ2 point mutation was expressed in plasmid-transfected CV-1 cells.

^**b**^ Ascertained by co-immunostaining with for μ2 with a specific mAb anti-HA and MT with anti-tubulin-Alexa 488.

^**c**^ Ascertained by co-expression of μNS and μ2-HA, followed by immunostaining with μNS-specific polyclonal antibodies and HA-specific mAb. Morphology of the FLS is also indicated: F, filamentous; G, globular.

^**d**^ Ascertained by (283–325)μ2 region fused to cytoplasmic platform assay as described in the text.

NT, not tested.

### μ2 self-association in a cytoplasmic platform assay and point mutations that abrogate it

**Fig 6:**
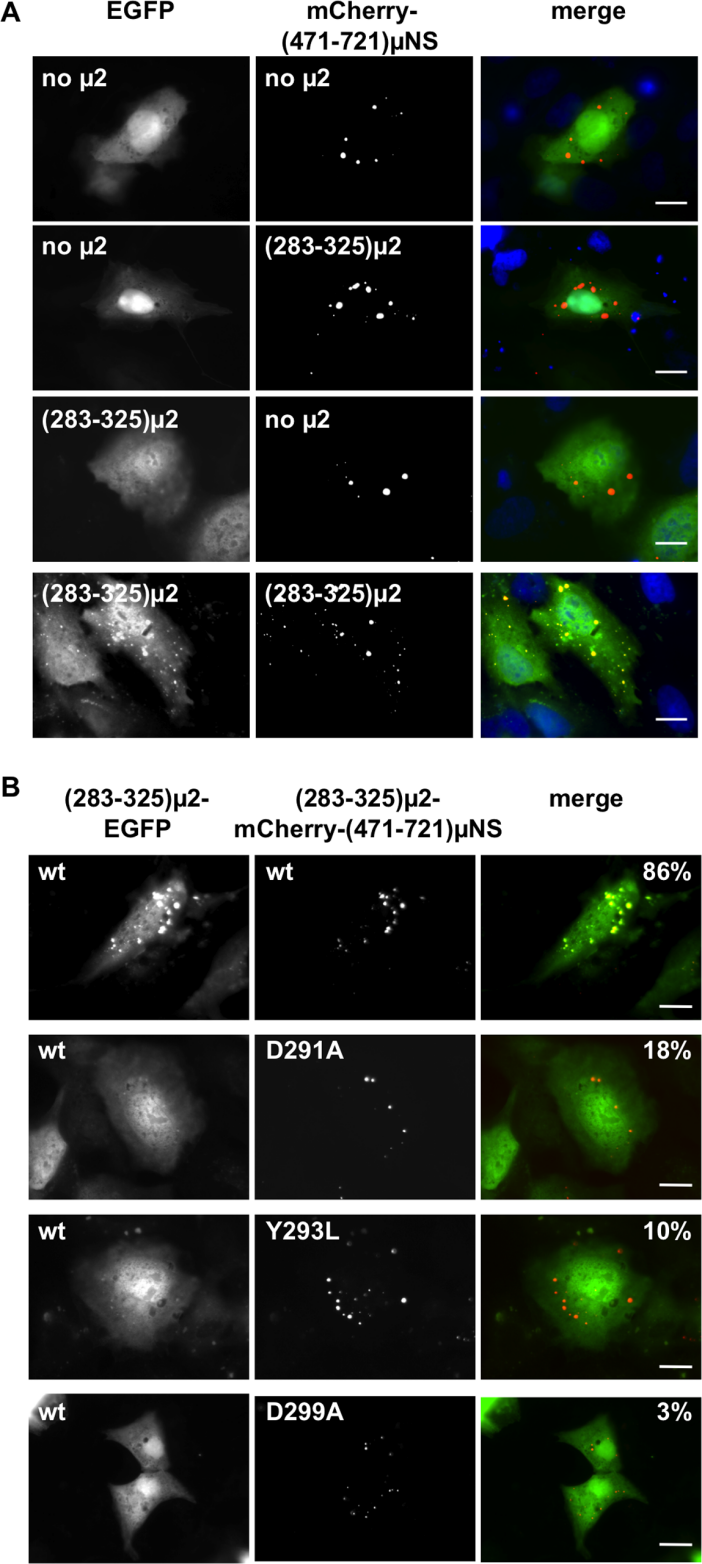
The amino acids D291, Y293 and D299 present in μ2 region from 283 to 325 are required for μ2 self-association. An *in vivo* protein interaction platform was performed, in which EGFP-tagged protein is "fish" and mCherry-tagged protein is "bait." (A) At 20 hpt, CV-1 cells co-expressing either EGFP (first and second rows) or (283–325)μ2-EGFP (third and fourth rows) with either mCherry-(471–721)μNS (first and third rows) or (283–325)μ2-mCherry-(471–721)μNS (second and fourth rows) were fixed and analyzed by fluorescence microscopy. A merged image is shown in the right column. Scale bar is 10 μm. (B) At 20 hpt, CV-1 cells co-expressing the “fish” (283–325)μ2-EGFP with the “bait” (283–325)μ2-mCherry-(471–721)μNS, respectively. (283–325)μ2-mCherry-(471–721)μNS containing μ2 wild-type or its individual point mutations are specified in the left top corner of each picture from the middle column. Cells were fixed and visualized by fluorescence microscopy. A merged picture is shown in the right column (EGFP, green; mCherry, red and nuclei stained with DAPI, blue). Scale bar is 10 μm.

We next tested directly whether μ2 residues 283–325 are involved in μ2 self-association. Previous studies have validated and shown the usefulness of (471–721)μNS as a platform for probing protein-protein associations in large cytoplasmic FLS [47–50, 59]. For this purpose, we fused the μ2 residues from region 283–325 to both the N-terminus of EGFP as well as to an mCherry-(471–721)μNS cytosolic platform. As observed in Fig 6A, when co-expressing EGFP with mCherry-(471–721)μNS (Fig 6A, first row) or both carrying a (283–325)μ2 region independently were unable to co-localize in the cytosolic platform (Fig 6A, second and third rows). However, when co-expressing (283–325)μ2-EGFP with (283–325)μ2-mCherry-(471–721)μNS (Fig 6A, fourth row), a positive cytosolic FLS platform co-localization was observed. Taken together, μ2 aa residues 283–325 resulted being sufficient for a self-association activity.

As mentioned above, point mutations in D291A, Y293L, and D299A of μ2 were sufficient to abrogate the MT-association (Fig 5), suggesting that one possibility is that these residues affect μ2 self-association. Under those circumstances, one would expect that mutations in these residues would also disrupt association by the 283–325 region in the platform assay. Indeed, when these mutations were tested, all three abrogated co-localization in the platform assay when present in either one of the co-expression partners or both (Fig 6B and S4 Fig).

These results provide further evidence for the involvement of the 283–325 region in a μ2 self-association activity. Notably, the point mutations D291A, Y293L, and D299A are involved in the abrogation of both MT-association and μ2-self-association.

### μ2 self-association involving the 283–325 region is important for MT-association

We took further advantage of the three point mutations D291A, Y293L, and D299A to address whether the μ2 self-association involving the 283–325 region is only important for μ2 association with MTs or whether the μ2 self-association involving other regions might be sufficient. To address this question, we co-expressed μ2-EGFP, as a known MT-associating protein (Fig 2), with μ2-HA, either with or without the point mutations in the latter. Although these mutations abrogate association with MTs by μ2-HA when expressed alone (Fig 5), the mutant proteins might associate with MTs in this co-expression setting if they can form functional hetero-oligomers with μ2-EGFP. On the other hand, if the self-association mediated by the 283–325 region is important for MT-association or is the only means of μ2 self-association, then these mutant proteins would remain negative for MT association even in the presence of μ2-EGFP.

**Fig 7:**
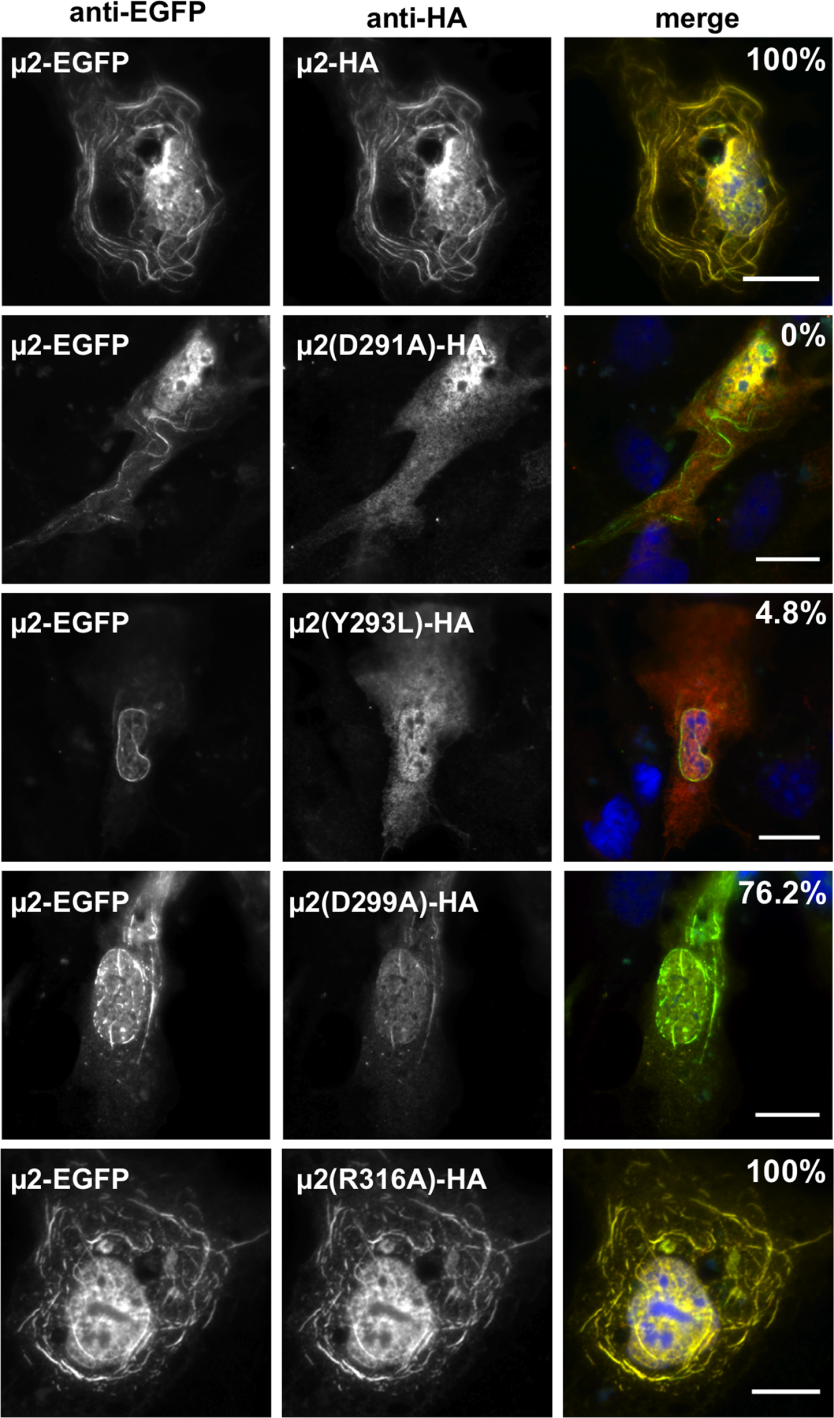
Amino acid residues Asp 291, Tyr 293 and Asp 299 abrogate μ2 self-association. (**A**) Immunofluorescence of CV-1 cells co-expressing both μ2 wild type fused to EGFP and μ2 wild type or containing the specified point mutation tagged to HA. At 20 hpt, cells were methanol fixed and immunostained for the visualization of μ2-EGFP (specific anti-EGFP serum, green) (left column) and μ2-HA and individual point mutations (mAb anti-HA, red)(middle column). Merged images are shown in the right column. Nuclei are stained with DAPI (blue). Scale bar is 10 μm. The percentage of positive cells co-expressing μ2-HA and μ2-EGFP, which form filamentous structures when associating is indicated in the top-right merged images.

To begin these experiments, μ2-EGFP and μ2-HA were co-expressed and found to co-localize on MTs (Fig 7A, first row), as expected from our previous results. When μ2-EGFP was co-expressed with μ2(D291A)-HA or μ2(Y293L)-HA, however, there was little or no colocalization, and the HA-tagged mutants remained diffusely distributed (Fig 7A, second and third rows). Interestingly, when μ2-EGFP was co-expressed with μ2(D299A)-HA, only weak colocalization on MTs was observed, but in a majority of the cells (Fig 7A, fourth row). When μ2-EGFP was co-expressed with μ2(R316A)-HA, as a control for a mutant that retains MT-association activity on its own (Fig 5 and Table 1), strong co-localization on MTs was again observed, comparable to that seen with μ2-HA (Fig 7). These results provide further evidence that μ2 residues Asp291 and Tyr293 are important for self-association involving the 283–325 region of μ2. Residue Asp299 appears to be somewhat less important in this respect since the D299A mutation allowed consistently weak colocalization in this assay.

Interestingly, these results indicate that μ2 self-association involving the 283–325 region is specifically important for MT-association. Thus, in the absence self-association ability, the mutation-containing μ2-HA proteins not only failed to associate with MTs on their own **(Fig 5)** but also failed to be recruited to MTs by μ2 *in trans* (Fig 7).

### μ2 region necessary and sufficient for the association with matrix protein μNS in FLS

**Fig 8:**
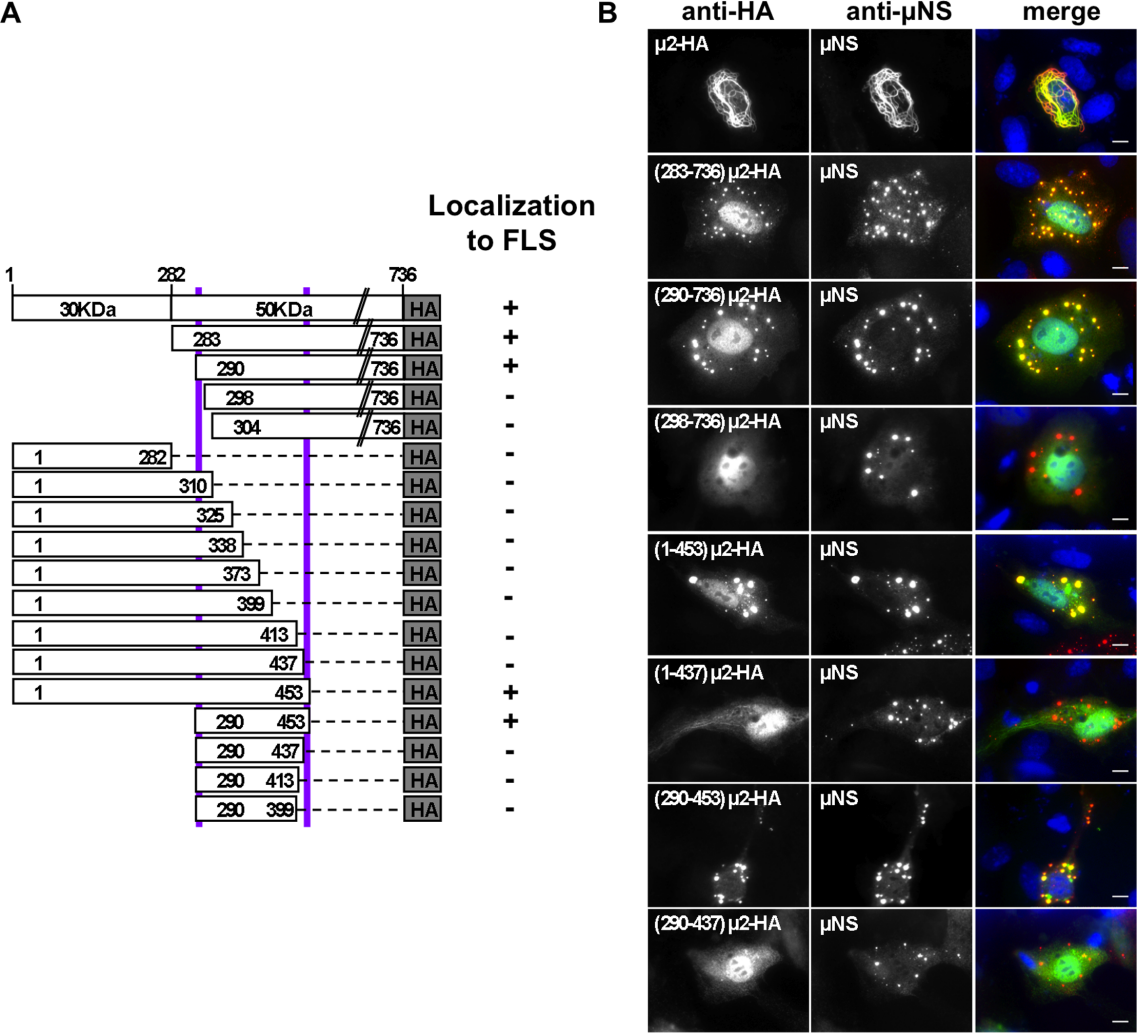
μ2 region necessary and sufficient for μNS association in FLS. (A) Schematic representation of T1L μ2 deletion mutants fused to an HA tag (not to scale). Positive (+) or negative (-) co-localization to μNS-FLS phenotype is indicated at right. (B) Most representative immunofluorescence of CV-1 cells co-expressing the indicated μ2 deletion mutant HA-tagged along with T1L μNS. At 20 hpt, cells were fixed and immunostained for detection of μ2 (mouse mAb anti-HA, green) (left column) and μNS (specific anti-μNS serum, red)(middle column). A merged image is shown in the right column. Nuclei are stained with DAPI (blue). Scale bar is 10 μm.

As shown above, when the 50K region of μ2 was fused to EGFP it was unable to associate to MTs, but instead, it associates to μNS by co-localizing in FLS **(Fig 2)**. To investigate which is the minimal region of μ2 necessary and sufficient for the association to μNS in FLS, we sequentially deleted μ2 from its N-and C-terminus, fusing an HA-tag at the C-terminal of each deletion mutant (Fig 8A). These μ2 deletion mutants were analyzed for their capacity to associate with μNS in FLS. As expected, μ2 full-length-HA and deletion mutant (283–736)μ2-HA associate with μNS when co-expressed, forming filamentous and globular FLS respectively (Fig 8B, first and second rows). As anticipated, the 30K region of the μ2 construct (1–282)μ2-HA could not localize in globular μNS FLS (Fig 8). We then systematically deleted the N-terminus of μ2, checking for its ability for μNS-FLS localization. Our data show that making a deletion from residue 290 the capacity to localize in globular μNS-FLS is retained, but this ability is lost when deleting up to residue 298 (Fig 8 and S5 Fig). Next, we engineered μ2 constructions with sequential deletions from its C-terminus. From these constructions, our results indicate that the μ2 up to residue 453 was necessary to retain the capacity to localize in globular μNS-FLS (Fig 8). We then tested a shorter μ2 construction, including residues 290 to 453, resulted in a positive co-localization to μNS-FLS. This outcome was, in fact, the minimal region necessary and sufficient of μ2 required to associate with μNS in FLS, since shorter versions, like (290–437)μ2-HA, were unable to localize in μNS-FLS remaining diffuse in the cytosol (Fig 8 and S5 Fig). Notably, the construction 290 to 453 also encompasses the μ2 self-association region.

## Discussion

The lack of an X-ray crystal structure for the MRV μ2 protein, the only MRV structural protein for which this restriction remains [4, 8, 60–62], has made it difficult to define the regions involved in its activities, which include RNA binding [21]; hydrolysis of the γ-phosphate from NTPs and RNA 5´-termini [13, 15]; and associations with λ3 [13], μNS [24], and MTs [13, 23]. A better understanding of these activities should shed light on the roles of μ2 in the MRV life cycle and its effects on cells, e.g., as a critical component of the transcriptase and replicase complexes [16, 18, 63] and viral factories [14, 22, 23, 29, 30]. Of particular interest is the basis for genetic influences of M1/μ2 on MRV growth in different cells and in MRV growth and virulence in animals [33, 35, 39, 40, 41, 42].

**Fig 9:**
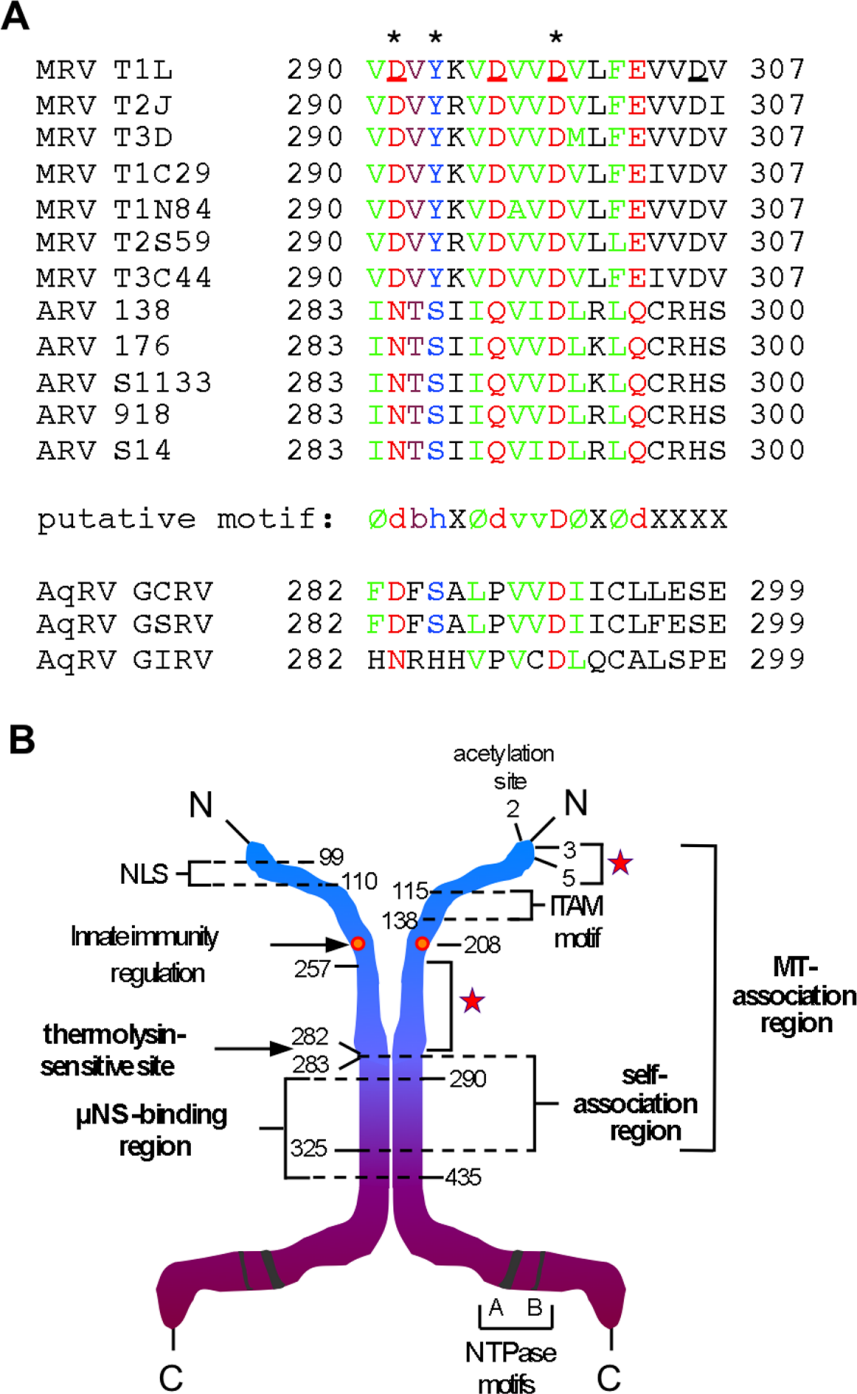
Self-association motifs sequence and model for μ2 binding regions. (A) T-Coffee alignment of μ2-homolog proteins in the 290–307 region of μ2. GenBank accession numbers are listed in Materials and Methods. T2J, Type 2 Jones; T1C29, Type 1 Clone 29; T1N84, Type 1 Netherlands 1984; T2S59, Type 2 Simian Virus 59; T3C44, Type 3 clone 44; GCRV, Grass carp reovirus; GSRV, Golden shiner reovirus; GIRV, Golden ide reovirus; see text for other abbreviations. Members of the genus *Orthoreovirus*, MRVs and avian reoviruses (ARVs), are listed above the putative sequence motif defined by the MRV and ARV sequences. Members of the related genus *Aquareovirus*, aquareoviruses (AqRVs), are listed below the motif. In the motif, Ø means hydrophobic residue (A, F, I, L, M, V) (letters in blue-green), d means acid or amide residues (D, E, N, Q) (letters in orange), b means b-branching residues (T, V) (letters in purple), h means hydroxylated residues (S, Y) (letters in yellow-green), X means variable residues (letters in black), v means mostly conserved Val residue (letters in blue-green), and D means conserved Asp residue (letters in orange). Shading identifies residues that vary from the consensus within each group (MRVs, ARVs, and AqRVs). Residues shown by point mutagenesis to be important for μ2 self-association and microtubule association in this study are indicated above by (*). (B) Summary diagram of MRV μ2. The 736-aa μ2 protein is represented as a dimer, having separate N- and C-terminal domains and a central region of self-association. The putative N-terminal domain (residues 1–282), acetylated at Ala2, is shown in blue, and the putative C-terminal domain (residues 283–736) is shown in purple. The unique protease-hypersensitive region is found between these two domains, N-terminally abutting the self-association region. The self-association region might represent a separate, central domain. Residues 1–325 were shown to be the minimal contiguous region for MT-association in cells, as labeled. Three smaller regions were determined to be important for MT-association. Two of these regions, residues 3–5 and 257–282, are each indicated by a red star; the third, residues 283–325, is also necessary for μ2 self-association, as labeled. Mutation P208S, previously related to loss of MT-association and innate immunity regulation [23], is indicated by an orange dot. Residues 99–110[14] and 115–118 [32] are described as sufficient for μ2 nuclear localization (NLS) and as related to immunoreceptor tyrosine-based activation motif (ITAM) signaling cascade, respectively. The two previously identified NTPase/RTPase motifs, A (residues 410–420) and B (residues 446–449) [15, 70], are also indicated. The association region for MRV factory-matrix protein μNS has been mapped between residues 290 and 399.

In this study, we made a dissection of several specific activities of μ2. We began by exploring its association with MTs in cells and, through those studies, we were led to investigate a newly identified activity, i.e., the self-association involving the aa 290–325 region. The new evidence for μ2 self-association is not surprising. Preliminary results (X. Lu, J.K., M.L.N., and S.C. Harrison; also see reference [13]) have suggested that μ2 takes the form of a dimer or other small oligomer upon purification. Moreover, stoichiometric determinations from protein gels of MRV particles have suggested that there are ~20 copies of μ2 per virion or core [5], consistent with two copies of μ2 for either each of the ten genome segments (20 copies) or each of the twelve icosahedral fivefold vertices (24 copies). Evidence for localization of μ2 to the vertices has come from a reduction in the intensity of the μ2 protein band in combination with a decrease in the density of inwardly projecting transcriptase complexes near the fivefold axes of protease-treated, genome-deficient particles [63]. The fivefold axes are where λ3 (the viral RdRp) is located [64], so the μ2-λ3 interaction shown with purified proteins [13] is consistent with this location of μ2. Whether self-association is essential for the functions of μ2 in cores (NTPase, RTPase, putative RNA helicase, a putative cofactor for the viral RdRp in transcriptase and replicase complexes) [13, 15, 16, 18] remains yet to be elucidated. The self-association activity was mapped by deletion mutations to the μ2 residues 283–325, with point mutations assigning specific importance to Asp291, Tyr293, and Asp299. Notably, the μ2 homolog protein from avian orthoreovirus, μA, shows a strong degree of sequence conservation with μ2 residues 290–307 (Fig 9A), suggesting that self-association may be common to many or all members of the genus *Orthoreovirus*. In the case of VP5, the μ2 homolog protein from aquareoviruses, there is sequence conservation with μ2 residues 290–307 as well (Fig 9A), suggesting that members of the related genus *Aquareovirus* may also share this self-association activity. Of notice is the fact that self-association has not yet been experimentally demonstrated for these μ2 homologs of MRV relatives. To our knowledge, among ortho- and aquareoviruses, only MRVs have so far been shown to have MT-anchored viral factories [23, 26, 27, 29, 65–67], suggesting that any self-association by μ2 homologs μA and VP5 may have evolved and been maintained for reasons other than for MT-association, such as for the NTPase/RTPase activity or related functions [68, 69].

In this study, only the 283–325 region was shown to be sufficient for μ2 self-association, as reflected in the model diagram (Fig 9B). But might other regions of μ2 be in and/or sufficient for this activity as well? Our experiments to date have not addressed this question, although we cannot exclude that other additional μ2 activities require an oligomeric conformation, as is the case for nuclear transport [14] or regulation of host innate immune response mediated through an ITAM motif [32]. Since association with μNS by any of the three point mutations that abrogate association with both MTs and self (Table 1), self-association involving the 283–325 region of μ2 appears not to be essential for the μNS association.

The new evidence that an N-terminal region of μ2 is necessary and sufficient for MT association is also not surprising. In particular, previous results have shown that point mutation P208S reduces MT-association, such as in our lab’s version of MRV strain T3D [23, 29, 30]. This mutation appears to promote temperature-dependent misfolding of μ2 protein, with a subsequent increase in aggregation and polyubiquitination, along with reduced MT-association [30]. Lowered expression temperature (31 vs. 37°C) partially restores MT-association activity and reduces aggregation and polyubiquitination of P208S-containing μ2 [30]. Perhaps this folding defect is limited to the N-terminal, MT-binding domain of μ2 (Fig 9B), being other parts and functions of μ2 remaining intact. An important question is how self-association involving the 283–325 region of μ2 can be substantial for MT-association. In this case, the self-association involving some portion of residues 283–325 might contribute to forming a basal oligomer of μ2, such as a dimer as modeled in Fig 9B. This self-association would be important for stabilizing the MT-association, perhaps by allowing interaction of each μ2 oligomer to bind with MTs bundles. The self-association involving some portion of residues 283–325 might instead also represent a higher-order association between basal oligomers of μ2 that is relevant for stabilizing the association of μ2 oligomers playing a role in stabilizing MT-association, either within the same MT or MT-bundles. The observation that a weakly self-associating tag, specifically EGFP, can substitute for the 283–325 region of μ2 in promoting MT-association (Figs 2 and 4) is consistent with either of the proposed explanations.

There are many different kinds of cellular MT-associated proteins (MAPs), including the MAP1 and MAP2/Tau families (reviewed in [71] and [72]). These various families have different sequence requirements for binding to MTs. Many MAPs have hydrophobic regions involved in MT association, and the 164–282 region of μ2 is also relatively hydrophobic [29]. Some MAPs undergo post-transcriptional modification to enhance their binding to MTs, such as phosphorylation of MAP1B (reviewed in reference [73]). Preliminary data, however, indicate that μ2 is not phosphorylated, suggesting that this kind of signal transduction not be involved in the association between μ2 and MTs.

We also dissected the association of μ2 in FLS with MRV factory-matrix protein μNS, defined as the μ2 region 290–453. Notably, this region overlaps with part of the conserved μ2 self-association domain (aa 290–307) suggesting a tight regulation regarding the viral factory morphologies. As discussed above, the μNS association to μ2 is not impaired by disruptive self-association point mutations (D291A, Y293L, and D299A). On the same lines, previous evidence has shown that the morphology of FLS obtained after co-expression of μ2 and μNS is dependent upon the expression ratios of μ2 and μNS, with higher μ2 or μNS ratios forming filamentous or globular FLS, respectively [24]. This pattern can be observed during viral factories dynamics from the filamentous strain (like MRV T1L), in which at early and late times post-infection factories are seen as globular or filamentous, respectively. This effect also correlates with higher expression levels of μNS at early times post-infection and with increasing expression levels of μ2 at later times post-infection. In our model, we propose that μNS binds μ2 at region 290–453 at early times post-infection constituting globular viral factories and then at later times post-infection, the increased amounts of μ2 leads to its oligomerization allowing the MT binding and stabilization, which in turn form filamentous viral factories. Based on these data, we predict that over-expressing a μ2 dominant-negative carrying a point mutation in D291A, Y293L or D299A will lead to the generation of globular viral factories filamentous strain. Our data also suggest that a soluble portion of both μ2 and μNS can interact in the cytosol in the absence of FLS. This result is not surprising since it has been described for other members of the *Reoviridae* family, such as the association of the rotavirus proteins NSP2 and NSP5 in the soluble cytosolic portion, independently of viroplasm assembly [74]. The implications of these soluble interactions are not addressed in this study.

By associating with and stabilizing MTs, as well as anchoring the viral factories to them in the case of most MRV strains, μ2 has the potential for many different types of effects on host cells and tissues. To name just a few broad examples, μ2 could affect functions of MTs in the transport of vacuoles and other cargo, maintenance, and organization of cellular organelles, cell polarity, and adherence, cell motility, ciliary function, mitosis, and/or cytokinesis. Thus, learning more about μ2-MT associations and secondary effects may be key to understanding the roles of μ2 in growth and virulence in infected animals.

## Supporting information

S1 Fig (**A**) Confocal immunofluorescence of CV-1 cells expressing EGFP-tagged full-length, aa regions 1–282 or 283–736 from T1L μ2. At 20 hpt, cells were methanol fixed and immunostained for the detection of μ2 (specific anti-EGFP serum, green) (left column) and acetylated-MTs (mAb anti-acetylated alpha tubulin, red)(middle column). A merged image is shown in the right column. Nuclei are stained with DAPI (blue). Scale bar is 20μm. (**B**) Immunofluorescence of CV-1 cells expressing EGFP-tagged full-length T1L μ2 or (1–282)μ2. At 23 hpt, cells were treated for 1 hour with 10μM nocodazole. Afterward, cells were methanol fixed and immunostained for the detection of μ2 (specific anti-EGFP serum, green)(left column) and MTs (mAbs anti-alpha tubulin, red). The merged images are shown in the right column. Nuclei are stained with DAPI (blue). Scale bar is 20μm.
(TIF)

S2 Fig Pull down assay of CV-1 lysates co-expressing histidine tagged μ2-EGFP deletions and μNS at a transfection ratio of 2:1. (**A**) At 15 hpt, cell cultures images were acquired at the fluorescent microscope. Scale bar is 20 μm. (**B**) Immunoblotting of pulled down samples (cellular extract (ce), flow through (ft) and elution (el)) from nickel resin. Previous to lysis, cells were DSP cross-linked. The membranes were incubated with anti-EGFP, and anti-μNS for the detection of μ2-EGFP-H_6_ derived proteins (upper panel) and μNS (lower panel), respectively. The red dots show the monomeric isoform of μ2-EGFP-H_6_ or its derived deleted proteins.
(TIF)

S3 Fig Immunofluorescence of CV-1 cells co-expressing T1L μ2-HA tag or the indicated point mutations with T1L μNS. At 20 hpt, cells were methanol fixed and immunostained for the detection of μ2 (mAb anti-HA, green) (left column) and μNS (specific anti-μNS serum, red)(middle column). The merged images are shown in the right column. Nuclei are stained with DAPI (blue). Scale bar is 10μm.
(TIF)

S4 Fig At 20 hpt, CV-1 cells co-expressing the "fish" (283–325)μ2-EGFP along with both (283–325)μ2-mCherry-(471–721)μNS (left panel) or mCherry-(471–721)μNS (right panel) as "baits," containing wild type-μ2 or its point mutations as labeled, respectively. Cells were fixed and directly visualized by fluorescence microscopy. A merged image is shown in each right column (EGFP (green), green; mCherry (red) Scale bar is 10 μm.
(TIF)

S5 Fig Immunofluorescence of CV-1 cells co-expressing T1L μ2-HA tag or deletion mutants with T1L μNS. At 20 hpt, cells were methanol fixed and immunostained for the detection of μ2 (mAb anti-HA, green) (left column) and μNS (specific anti-μNS serum, red)(middle column). The merged images are shown in the right column. Nuclei are stained with DAPI (blue). Scale bar is 10μm.
(TIF)

S1 Table Primers for the μ2 deletion mutants and for the cytoplasmic platform segments constructions. (DOCX)

S2 Table Oligonucleotides used to introduce point mutations in μ2-HA. (DOCX)

S1 File Supporting materials and methods. Plasmid construction.
(DOCX)
