# Supplementary figures and images for: Dissection of mammalian orthoreovirus µ2 reveals a self-associative domain required for binding to microtubules but not to factory matrix protein µNS
Source: PLoS One. 2017 Sep 7;12(9):e0184356. doi: 10.1371/journal.pone.0184356 (PMC5589220; doi:10.1371/journal.pone.0184356)

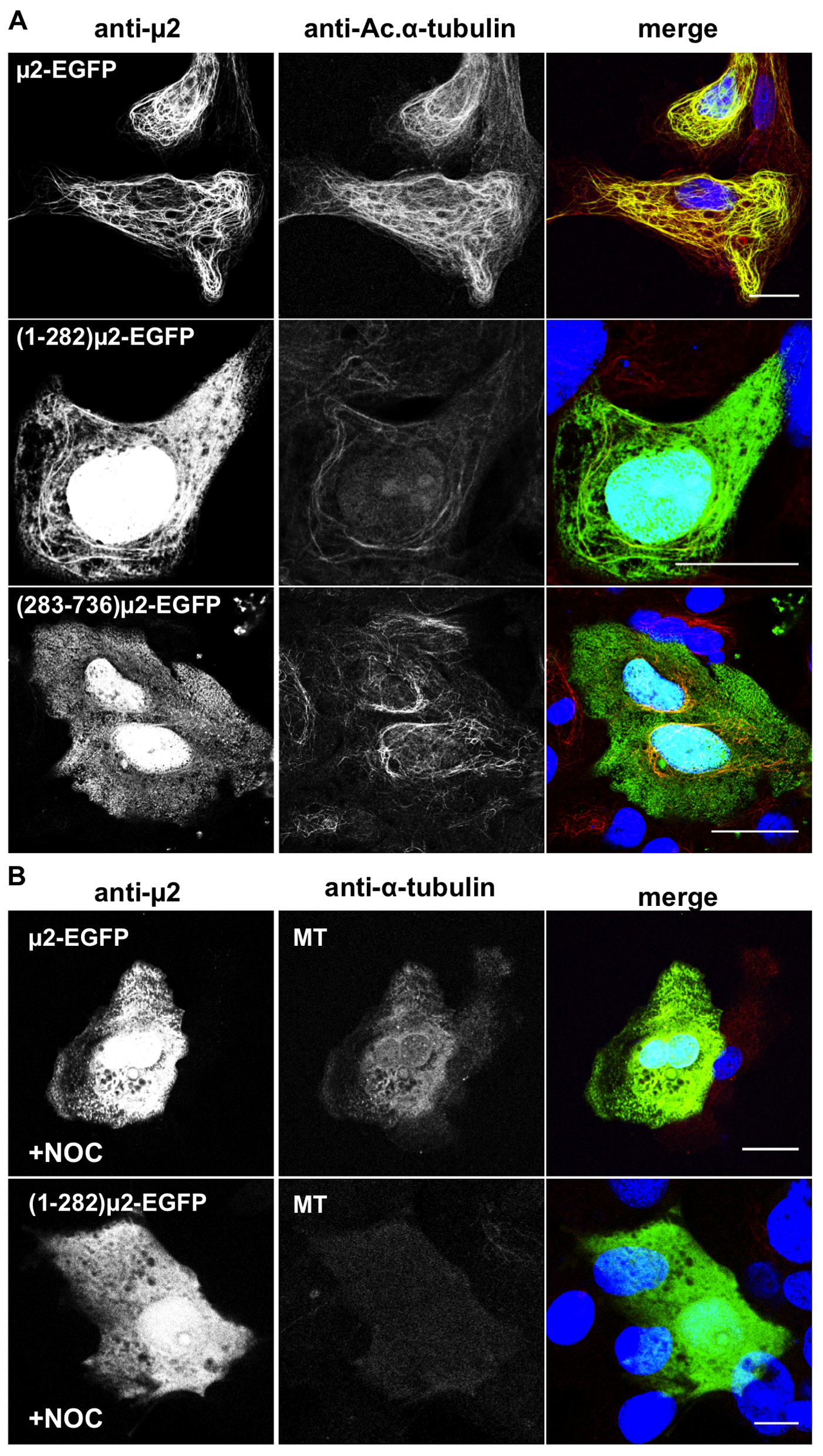

Supplement: S1 Fig — (A) Confocal immunofluorescence of CV-1 cells expressing EGFP-tagged full-length, aa regions 1–282 or 283–736 from T1L μ2. At 20 hpt, cells were methanol fixed and immunostained for the detection of μ2 (specific anti-EGFP serum, green) (left column) and acetylated-MTs (mAb anti-acetylated alpha tubulin, red)(middle column). A merged image is shown in the right column. Nuclei are stained with DAPI (blue). Scale bar is 20μm. (B) Immunofluorescence of CV-1 cells expressing EGFP-tagged full-length T1L μ2 or (1–282)μ2. At 23 hpt, cells were treated for 1 hour with 10μM nocodazole. Afterward, cells were methanol fixed and immunostained for the detection of μ2 (specific anti-EGFP serum, green)(left column) and MTs (mAbs anti-alpha tubulin, red). The merged images are shown in the right column. Nuclei are stained with DAPI (blue). Scale bar is 20μm. (TIF) [file pone.0184356.s001.tif]

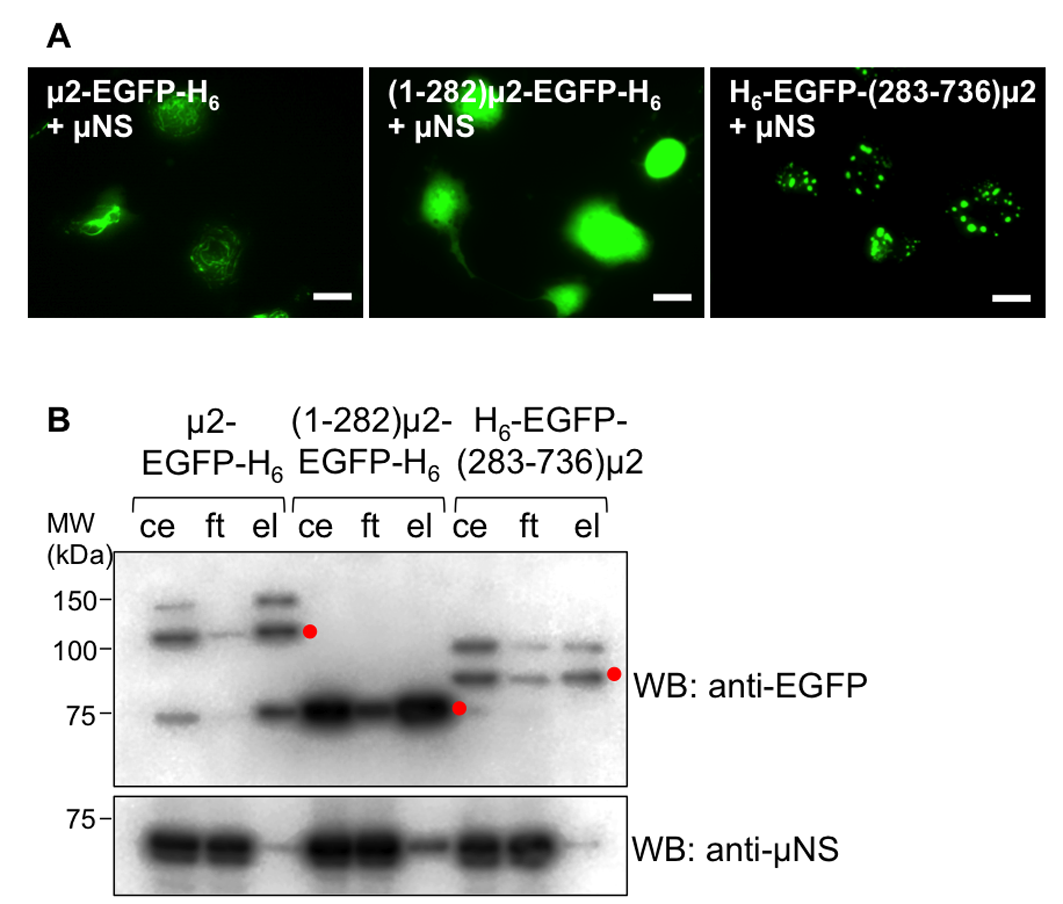

Supplement: S2 Fig — (A) At 15 hpt, cell cultures images were acquired at the fluorescent microscope. Scale bar is 20 μm. (B) Immunoblotting of pulled down samples (cellular extract (ce), flow through (ft) and elution (el)) from nickel resin. Previous to lysis, cells were DSP cross-linked. The membranes were incubated with anti-EGFP, and anti-μNS for the detection of μ2-EGFP-H6 derived proteins (upper panel) and μNS (lower panel), respectively. The red dots show the monomeric isoform of μ2-EGFP-H6 or its derived deleted proteins. (TIF) [file pone.0184356.s002.tif]

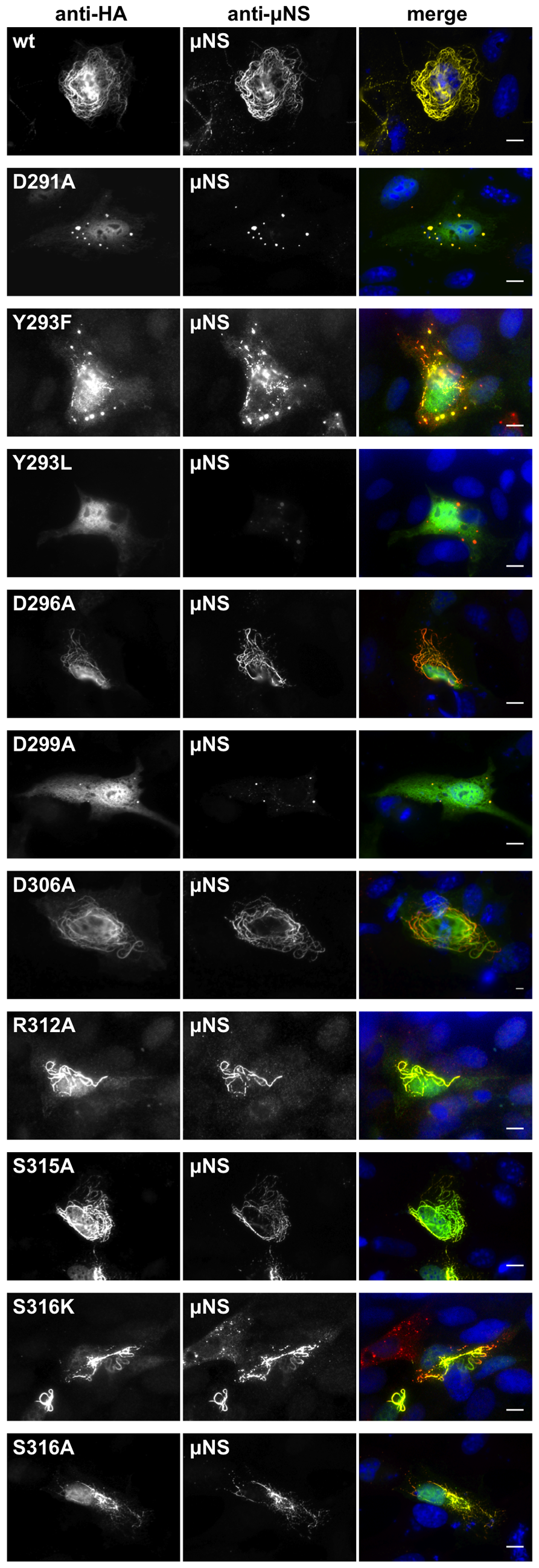

Supplement: S3 Fig — At 20 hpt, cells were methanol fixed and immunostained for the detection of μ2 (mAb anti-HA, green) (left column) and μNS (specific anti-μNS serum, red)(middle column). The merged images are shown in the right column. Nuclei are stained with DAPI (blue). Scale bar is 10μm. (TIF) [file pone.0184356.s003.tif]

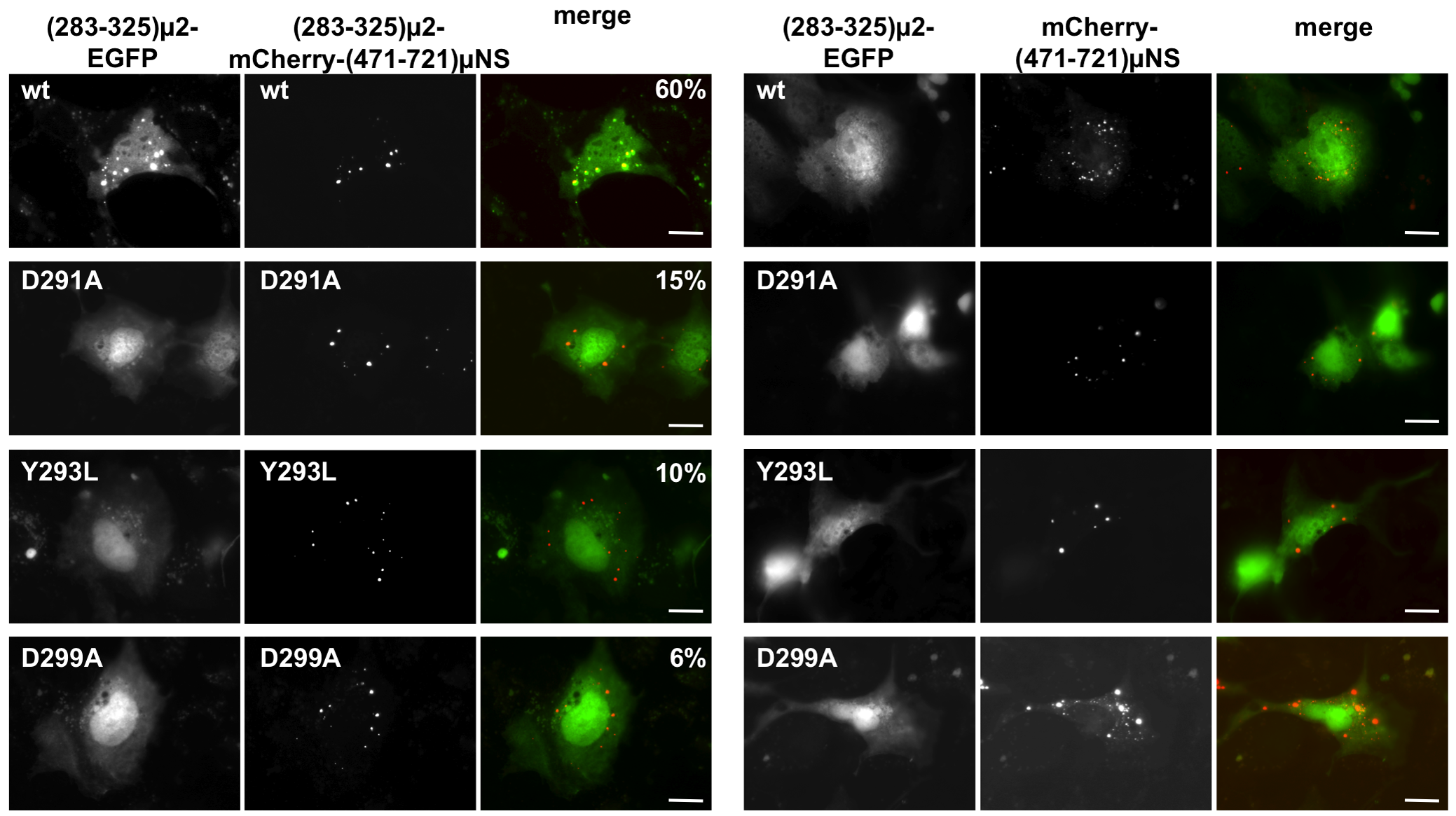

Supplement: S4 Fig — Cells were fixed and directly visualized by fluorescence microscopy. A merged image is shown in each right column (EGFP (green), green; mCherry (red) Scale bar is 10 μm. (TIF) [file pone.0184356.s004.tif]

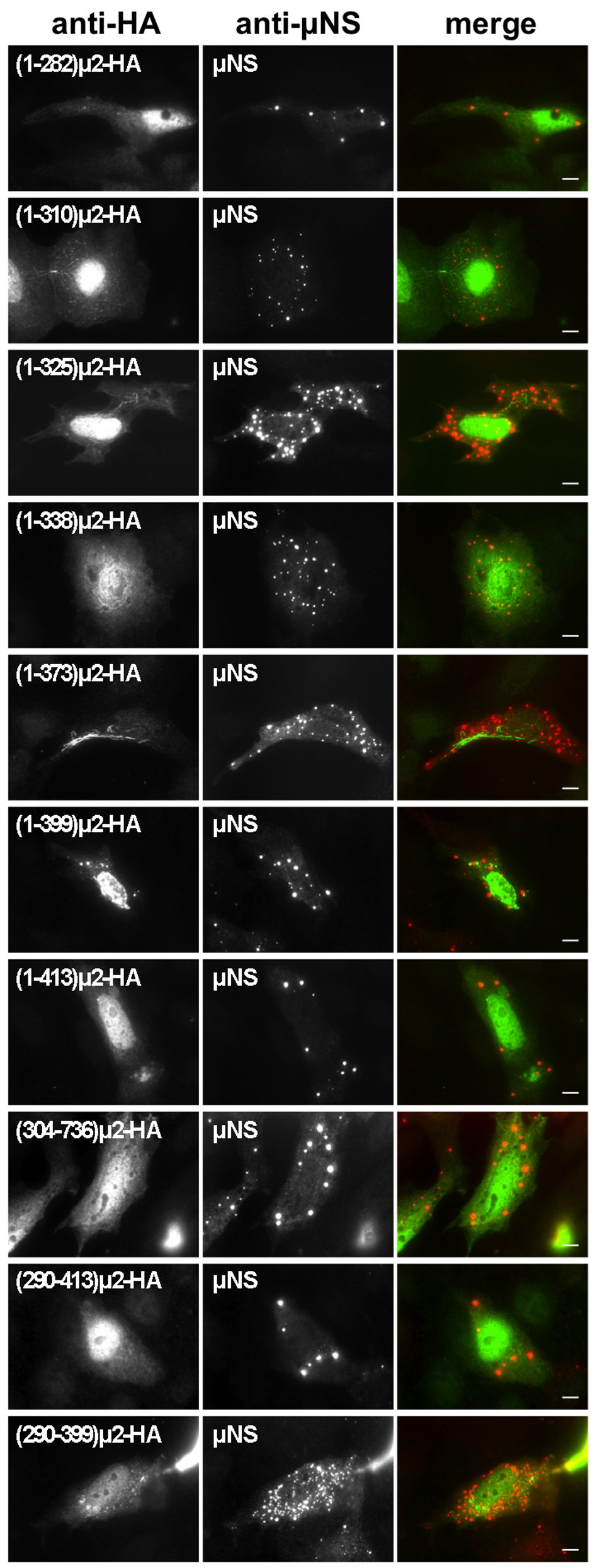

Supplement: S5 Fig — At 20 hpt, cells were methanol fixed and immunostained for the detection of μ2 (mAb anti-HA, green) (left column) and μNS (specific anti-μNS serum, red)(middle column). The merged images are shown in the right column. Nuclei are stained with DAPI (blue). Scale bar is 10μm. (TIF) [file pone.0184356.s005.tif]
