# Supplementary material for: Dissection of mammalian orthoreovirus µ2 reveals a self-associative domain required for binding to microtubules but not to factory matrix protein µNS
Source: PLoS One. 2017 Sep 7;12(9):e0184356. doi: 10.1371/journal.pone.0184356 (PMC5589220; doi:10.1371/journal.pone.0184356)
Supplement: S1 Table — (DOCX) [file pone.0184356.s006.docx]

### S1 Table. Primers for the µ2 deletion mutants and for the cytoplasmic platform segments constructions

| **Amplified**  **DNA segment** | **Oligonucleotide sequence** |
| --- | --- |
| (1-282)µ2-EGFP | Fwd.: 5’-ggggtaccaggcggca**atg**gcttacatcgcagttcc-3’ |
|  | Rev.: 5’-gggcggccgcttaatggtagggcaaatgcatc-3’ |
| EGFP-(283-736)µ2 | Fwd.: 5’-ggtgtacaaggcggcagttaaacgaggagcgtctc-3’ |
|  | Rev.: 5’-gggcggccgc**tta**cgccaagtcagatcggaaagc-3’ |
| (1-310)µ2-EGFP | Fwd.: 5’-gggaagcttgctattcgcggtcatggc-3’ |
|  | Rev.: 5’-ggggatccccatcggccacatccac-3’ |
| µ2-EGFP-H_6_ | Fwd.: 5’-gatcctcgag**atg**gcttacatcgcagttcct-3’  Rev.: 5’-gatcgcggccgc**tta**gtgatggtgatggtgatgtttctgtacag ctcgtccatgcc-3’ |
| (1-282)µ2-EGFP-H_6_ | Fwd.: 5’-gatcctcgag**atg**gcttacatcgcagttcct-3’  Rev.: 5’-gatcgcggccgc**tta**gtgatggtgatggtgatgtttctgtacag ctcgtccatgcc-3’ |
| H_6_-EGFP-(283-736)µ2 | Fwd.: 5’-gatcctcgag**atg**aaacatcaccatcaccatcacatggtgagca agggcgaggag-3’  Rev.: 5’-gatcgcggccgc**tca**cgccaagtcagatcggaaa-3’ |
| (1-338)µ2-HA | Fwd.: 5’-tagcctcgag**atg**gcttacatcgcagttcct-3’ |
|  | Rev.: 5’-gatcgcggccgc**tca**agcgtaatctggaacgtcgtatgggtac atgacatcgattgtatgcct-3’ |
| (1-373)µ2-HA | Fwd.: 5’-tagcctcgag**atg**gcttacatcgcagttcct-3’ |
|  | Rev.: 5’-gatcgcggccgc**tca**agctaatctggaacgtcgtatgggtaat aatcaagtattgaca-3’ |
| (1-325)µ2-HA | Fwd.: 5’-tagcctcgag**atg**gcttacatcgcagttcct-3’ |
|  | Rev.: 5’-gatcgcggccgc**tca**agcgtaatctggaacgtcgtatgggtaa tggtagggcaaatgcatcaa-3’ |
| mCherry | Fwd.: 5’-gatcgaattcgaattcggtaccatggtgagcaagggcgaggag gat-3’ |
|  | Rev.: 5’-gatcgcggccgc**tta**gttgtacagctcgtcgtccatgc-3’ |
| (471-721)µNS | Fwd.: 5’-gatctgtacaaggcagcgtccagtgacatggtagacggg-3’ |
|  | Rev.: 5’-gatcgcggccgc**tta**cagctcatcagttggaac-3’ |
| NSP5 | Fwd.: 5’-gatctgtacaagagtggaatgtctctca-3’ |
|  | Rev.: 5’-gatcgcggccgc**tta**caaatcttcgatcaattg-3’ |
| (283-736)µ2 ^a^ | Fwd.: 5’-gatcgctagc**atg**gttaaacgaggagcgtctcac-3’ |
|  | Rev.: 5’-gatcctcgagccgccaagtcagatcggaaagc-3’ |
| (283-736)µ2 ^b^ | Fwd.: 5’-gctaaccggt**atg**gttaaacgaggagcgtctcac-3’ |
|  | Rev.: 5’-gatcaccggtgccaagtcagatcggaaagc-3’ |
| (283-325)µ2 ^a^ | Fwd.: 5’-gatcgctagc**atg**gttaaacgaggagcgtctcac-3’ |
|  | Rev.: 5’-gatcctcgag**cta**ccggaacggtatgcatagc-3’ |
| (283-325)µ2 ^b^ | Fwd.: 5’-gctaaccggt**atg**gttaaacgaggagcgtctcac-3’ |
|  | Rev.: 5’-gatcaccggtaccggaacggtatgcatagt-3’ |

^a^ For fusion with mCherry in platform vectors

^b^ For fusion with EGFP in platform vectors

*Restriction enzymes sites are underlined

**Initiation and stop codons are labeled in bold
