# Supplementary material for: Dissection of mammalian orthoreovirus µ2 reveals a self-associative domain required for binding to microtubules but not to factory matrix protein µNS
Source: PLoS One. 2017 Sep 7;12(9):e0184356. doi: 10.1371/journal.pone.0184356 (PMC5589220; doi:10.1371/journal.pone.0184356)
Supplement: S2 Table — (DOCX) [file pone.0184356.s007.docx]

### S2 table. Oligonucleotides used to introduce point mutations in µ2-HA

| **Amplified DNA segment** | **Oligonucleotide sequence** |
| --- | --- |
| D291A | Fwd.: 5’-tctcacgtcgccgtttacaag-3’ |
|  | Rev.: 5’-cttgtaaacggcgacgtgaga-3’ |
| Y293F | Fwd.: 5’-cacgtcgacgttttcaaggtggatgtt-3’ |
|  | Rev.: 5’-aacatccaccttgaaaacgtcgacgtg-3’ |
| Y293L | Fwd.: 5’-cacgtcgacgttctcaaggtggatgtt-3’ |
|  | Rev.: 5’-aacatccaccttgagaacgtcgacgtg-3’ |
| D296A | Fwd.: 5’-tacaaggtggctgttgtagac-3’ |
|  | Rev.: 5’-gtctacaacagccaccttgta-3’ |
| D299A | Fwd.: 5’-gatgttgtagccgtgttgttc-3’ |
|  | Rev.: 5’-gaacaacacggctacaacatc-3’ |
| D306A | Fwd.: 5’-gaggtagtggctgtggccgat-3’ |
|  | Rev.: 5’-atcggccacagccactacctc-3’ |
| R312A | Fwd.: 5’-cgatgggttggccaacgtatct-3’ |
|  | Rev.: 5’-agatacgttggccaacccatcg-3’ |
| S315A | Fwd.: 5’-gcgcaacgtagctaggaaact-3’ |
|  | Rev.: 5’-agtttcctagctacgttgcgc-3’ |
| R316K | Fwd.: 5’-cgcaacgtatctaagaaactaactatg-3’ |
|  | Rev.: 5’-catagttagtttcttagatacgttgcg-3’ |
| R316A | Fwd.: 5’-caacgtatctgcgaaactaact-3’ |
|  | Rev.: 5’-agttagtttcgcagatacgttg-3´ |

^*^ Mutated nucleotides are underlined.
