# Supplementary material for: Dissection of mammalian orthoreovirus µ2 reveals a self-associative domain required for binding to microtubules but not to factory matrix protein µNS
Source: PLoS One. 2017 Sep 7;12(9):e0184356. doi: 10.1371/journal.pone.0184356 (PMC5589220; doi:10.1371/journal.pone.0184356)
Supplement: S1 File — Plasmid construction. (DOCX) [file pone.0184356.s008.docx]

## Supporting Materials and Methods

### Plasmid construction

Constructs pEGFP-µ2 and pEGFP-(1-282)µ2 were obtained by PCR amplification of the respective fragments from pBS-M1(T1L) [23], using specific primers to insert flanking *Acc*65I and *Not*I sites, followed by ligation into those sites in pEGFP-N1 (Invitrogen). Constructs pEGFP-(1-310)µ2, pEGFP-(1-257)µ2, pEGFP-(1-310∆3-17)µ2 and pEGFP-(1-310∆3-5)µ2 were obtained by PCR amplification of the respective fragments from pBS-M1(T1L), using specific primers to insert flanking *Hind*III and *Bam*HI sites, followed by ligation into those sites in pEGFP-N1 (Clontech). Construct pEGFP-(283-736)µ2 was obtained by PCR amplification of the indicated fragment from pBS-M1(T1L), using specific primers to insert flanking *Bsr*GI and *Not*I sites, followed by ligation into those sites in pEGFP-N1(Clontech). Constructs pCI-µ2-EGFP-H_6_ and pCI-(1-282)µ2-EGFP-H_6_ were obtained by PCR amplification of the respective fragments from pEGFP-µ2 and pEGFP-(1-282)µ2, using specific primers to insert *Xho*I and hexahistidine-tag/*Not*I sites followed by ligation in those sites in pCI-Neo (Promega). pCI-H_6_-EGFP-(283-736) was obtained by PCR amplification of pEGFP-(283-736)µ2 using specific primers to insert *Xho*I/hexahistidine tag and *Not*I sites followed by ligation in those sites in pCI-Neo (Promega).

Constructs pCI-µ2-HA, pCI-(1-338)µ2-HA, and pCI-(1-373)µ2-HA were obtained by PCR amplification of the respective fragments from pCI-M1(T1L) [23], using specific primers to insert flanking *Xho*I and HA-tag/*Not*I sites, followed by ligation into those sites in pCI-Neo (Promega). Construct pCI-(Xho/Age-HA-Not) was constructed by annealing the oligonucleotides 5´-TCGAGCCACCGGTATACCCATACGACGTTCCAGATTACGCTTGAGC-3´ and 5´-GGCCGCTCAAGCGTAATCTGGAACGTCGTATGGGTATACCGGTGGC-3´, followed by ligation into the *Xho*I and *Not*I sites of pCI-Neo. Constructs pCI-(1-282)µ2-HA and pCI-(1-310)µ2-HA were then obtained by *Xho*I/*Age*I digestion of pEGFP-(1-282)µ2 and pEGFP-(1-310)µ2, respectively, followed by ligation into those sites in pCI-(Xho/Age-HA-Not). Construct pCI-HA was constructed by annealing the oligonucleotides 5´-CGCGTGCTTACCCATA CGTTCCAGATTACGCTTGAGC-3´ and 5´-GGCCGCTCAAGCGTAATCTGGAACGTCGT ATGGGTAAGCA-3´, followed by ligation into the *Mlu*I and *Not*I sites of pCI-Neo. Construct pCI-(1-325)µ2-HA was then obtained by PCR amplification of the indicated fragment from pCI-M1(T1L), using specific primers to insert flanking *Xho*I and *Mlu*I sites, followed by ligation into those sites in pCI-HA. Construct pCI-(Xho-ATG-283 SalI/NotI)µ2 was obtained by annealing oligonucleotides 5´-TCGAGATGGTTAAACGAGGAGCGTCTCACGTCGACGTTGC-3´ and 5´-GGCCCAACGTAGACGTGAGACGCTCCTCGTTTAACCATC-3´, followed by ligation into the *Xho*I and *Not*I sites of pCI-Neo. Construct pCI-(283-736)µ2-HA was then obtained by *Sal*I/*Not*I digestion of pCI-µ2-HA, followed by ligation into those sites in pCI-(Xho-ATG-283 SalI/NotI)µ2.

Construct pCI-EGFP was achieved by ligation of the *Eco*RI/*Not*I fragment from pEGFP-N1 into those sites in pCI-Neo. Construct pCI-(283-325)µ2-EGFP, as well as versions of this plasmid containing the corresponding µ2 point mutations D291A, Y293L, D299A, and R316A, were obtained by PCR amplification of the respective pCI-µ2-HA construct, using specific primers to insert flanking *Xho*I and *Age*I sites and followed by ligation into those sites in pCI-EGFP. Constructs pCMV-(283-736)µ2-EGFP-NSP5 and pCMV-(283-325)µ2-EGFP-NSP5 were obtained by PCR amplification of the indicated fragments from pCI-M1(T1L), using specific primers to insert flanking *Age*I sites into pCMV-EGFP-NSP5 (gently donated by the Oscar Burrone lab). Construct pCI-mCherry was obtained by PCR amplification of the mCherry fragment from pRSET-B-mCherry [57] (gently donated by the Roger Tsien lab), using specific primers to insert flanking *Eco*RI and *Not*I sites, followed by ligation into those sites in pCI-Neo. Construct pCI-mCherry-(471-721)µNS vector was next obtained by PCR amplification of the (471-721)µNS fragment from pCI-M3(T1L) [46], using specific primers to insert flanking *Bsr*GI and *Not*I sites, followed by ligation into those sites in pCI-mCherry. Construct pCI-(283-325)µ2-mCherry-(471-721)µNS and the versions containing the µ2 point mutations D291A, Y293L, and D299A were obtained by PCR amplification of the corresponding pCI-µ2-HA construct, using specific primers to insert flanking *Nhe*I and *Xho*I sites and followed by ligation into those sites in pCI-mCherry-(471-721)µNS. All the oligonucleotides are listed in S1 Table.

Versions of construct pCI-µ2-HA containing the µ2 point mutations D291A, Y293F, Y293L, D296A, D299A, R312K, S315A or R316A were built by insertion of point mutations using the QuickChange site-directed mutagenesis kit and protocol (Stratagene) and primers listed S2 Table.
